# Factors influencing adolescent girls and young women’s participation in a combination HIV prevention intervention in South Africa

**DOI:** 10.1186/s12889-021-10462-z

**Published:** 2021-02-27

**Authors:** Tracy McClinton Appollis, Zoe Duby, Kim Jonas, Janan Dietrich, Kealeboga Maruping, Fareed Abdullah, Nevilene Slingers, Catherine Mathews

**Affiliations:** 1 Health Systems Research Unit, South African Medical Research Council, Tygerberg, PO Box 19070, Cape Town, 7505 South Africa; 2 Adolescent Health Research Unit, Division of Child & Adolescent Psychiatry, Department of Psychiatry and Mental Health, University of Cape Town, 46 Sawkins Road, Rondebosch, Cape Town, 7700 South Africa; 3 Division of Social and Behavioural Sciences in the School of Public Health and Family Medicine, University of Cape Town, Cape Town, South Africa; 4 Perinatal HIV Research Unit, School of Clinical Medicine, Faculty of Health Sciences, University of the Witwatersrand, Johannesburg, South Africa; 5 Office of AIDS and TB Research, South African Medical Research Council, Tygerberg, PO Box 19070, Cape Town, 7505 South Africa; 6 Department of Internal Medicine, University of Pretoria, Pretoria, South Africa

**Keywords:** Participation, Intervention, Recruitment, Retention, Girls, Women, South Africa, HIV

## Abstract

**Background:**

For interventions to reach those they are intended for, an understanding of the factors that influence their participation, as well as the facilitators and barriers of participation are needed. This study explores factors associated with participation in a combination HIV prevention intervention targeting adolescent girls and young women (AGYW) aged 15–24-years-old, as well as the perspectives of AGYW, intervention implementers, and facilitators who participated in this intervention.

**Methods:**

This study used mixed-methods approach with quantitative household survey data from 4399 AGYW aged 15–24-years-old in six of the ten districts in which the intervention was implemented. In addition, qualitative methods included a total of 100 semi-structured in-depth interviews and 21 focus group discussions in five of the ten intervention districts with 185 AGYW who participated in one or more of the key components of the intervention, and 13 intervention implementers and 13 facilitators. Thematic analysis was used to explore the perspectives of participating and implementing the intervention.

**Results:**

Findings reveal that almost half of AGYW (48.4%) living in the districts where the intervention took place, participated in at least one of the components of the intervention. For both 15–19-year-olds and 20–24-year-olds, factors associated with increased participation in the intervention included being HIV negative, in school, never been pregnant, and having had a boyfriend. Experiencing intimate partner violence (IPV) and/or sexual violence in the past 12 months was associated with increased levels of participation in the intervention for 20–24-year-olds only. In our analysis of the qualitative data, facilitators to participation included motivating participants to join the interventions through explaining the benefits of the programme. Barriers included misguided expectations about financial rewards or job opportunities; competing responsibilities, interests or activities; family responsibilities including childcare; inappropriate incentives; inability to disrupt the school curriculum and difficulties with conducting interventions after school hours due to safety concerns; miscommunication about meetings; as well as struggles to reach out-of-school AGYW.

**Conclusion:**

Designers of combination HIV prevention interventions need to address the barriers to participation so that AGYW can attend without risking their safety and compromising their family, childcare and schooling responsibilities. Strategies to create demand need to include clear communication about the nature and potential benefits of such interventions, and the inclusion of valued incentives.

**Supplementary Information:**

The online version contains supplementary material available at 10.1186/s12889-021-10462-z.

## Background

Adolescent girls and young women (AGYW) aged 15–24 years make up only 10% of the population in sub-Saharan Africa, however they accounted for one in five new HIV infections in 2017 [1]. HIV and AIDS accounts for 71% of the global burden in sub-Saharan Africa with 18% of people living with HIV estimated to live in South Africa. Teenage pregnancy is common among South African youth [2] and may lead to poor health outcomes, poverty, [3] and school drop-out [4].

Adolescents in South Africa and elsewhere in the world require access to evidence-based interventions to prevent and manage HIV and unintended pregnancy. However, young people face barriers accessing sexual and reproductive health (SRH) interventions at government public sector health care facilities including cost [5], transportation, clinic hours [6], privacy and confidentiality, lack of adolescent and youth friendly health services in primary health care facilities [7], and negative health worker attitudes [8]. To reach adolescents and youth, interventions should also be made available outside of the context of government health care services. Out-of-health facility approaches involve service provision at places where adolescents live and congregate, such as in communities and schools.

Interventions in communities and schools are important because many of the factors that contribute to HIV and unintended pregnancy among AGYW lie outside of the health sector. For example, a supportive social environment for adolescents’ SRH service use, high quality education, employment opportunities, and the absence of interpersonal violence are at least as important as health service provision [9]. A holistic approach to adolescent and youth health therefore implies intersectoral action [10].

The concept of “combination implementation” has been defined as “the pragmatic, localized application of a package of evidence-based prevention interventions using optimized implementation and operational strategies to achieve high sustained uptake of good quality services” [11]. One of the key strategic objectives of the South African National Strategic Plan for HIV, STI’s and TB (2017–2022), was to develop combination interventions for the prevention of new HIV and sexually transmitted infections (STIs) [12]. From 2016 to the present day, the Global Fund to Fight AIDS, TB and Malaria has invested in a combination HIV prevention intervention for adolescent girls and young women in South Africa, which aims to reduce HIV incidence, teenage pregnancy, gender-based violence and to increase retention in schools, and access to economic opportunities. The intervention is delivered by various South African government and non-government organisations, and comprises a comprehensive package of health, education, and support services for delivery through schools and in communities within 10 South African districts. These districts were selected based on a determination that the AGYW living in them were among those with the highest HIV incidence in South Africa.

There is wide consensus that to achieve universal health coverage (UHC), people who need a service should obtain it in a timely manner and at a level of quality necessary to achieving the desired effect and potential health gains [13]. The concept of “effective coverage” (EC) refers to the proportion of a population in need of a service that experienced a positive health outcome from the service [14]. EC can be measured using health service coverage cascades applied to a clearly defined target population, for example AGYW with a specific health need, and including successive measures of contact with the health service/intervention, readiness of health service/intervention to deliver the service, receipt of appropriate and timely care, user-adherence, user-experience of care, disease control or prevention, well-being, health and survival [14]. The first and necessary step in achieving effective coverage has been described as “contact coverage” [15] or “service contact coverage*”* [14] and refers to the proportion of the population in need who come into contact with the (relevant) health service or intervention (also described as contact between the service provider and the user).

In the context of combination HIV prevention interventions for AGYW, there are a range of barriers that might prevent intervention contact coverage. For example, the difficulties of reaching school-going adolescents with after-school HIV prevention and SRH promotion interventions have been described in one South African study, in which it was found that adolescents who needed the intervention most were least likely to participate and attend sessions [16].

Using data from the HERStory Study, an evaluation of the South African AGYW combination intervention, this study describes intervention contact coverage: the extent to which AGYWs living in the intervention districts were reached and participated in, and were thus “covered” by the key intervention components. Assuming all AGYW in the intervention districts were “in need” of the intervention, it provides a description of the proportion of AGYW in the intervention districts who were recruited into, and who participated in at least one of the key components of the combination intervention, and the factors associated with participation, disaggregated by age. Furthermore, it describes AGYW’s perspectives of being recruited into and participating in the intervention, and intervention implementers’ and facilitators’ perspectives of recruiting and retaining AGYW in the intervention.

## Methods

### Intervention background

The Global Fund (GF) invested in a combination HIV prevention intervention for AGYW aged 10–24 years from 2016 to 2019. The combination intervention for AGYW was implemented in 10 South African districts. Key services and commodities were delivered through clubs and a “Keeping Girls in School programme” (KGIS). The clubs and programmes had different target groups (Table 1). The KGIS programme was a school-based programme which provided comprehensive SRH education, referrals to HIV testing and TB screening, linkage to care for HIV, pregnancy and other conditions, career guidance and homework support to encourage school attendance and completion of high school. Soul Buddyz Clubs (SBC) were aimed at raising awareness of various health issues, including life skills related to HIV/AIDS, relationships and sexuality, as well as promoting prevention of violence and alcohol and drug abuse. RISE or Women of Worth (WOW) clubs, aimed to build the resilience of young women and link them to biomedical services such as HIV Testing Services (HTS), prevention of mother-to-child transmission, antiretroviral therapy, modern contraceptives other SRH services, as well as provided economic strengthening and SRH education.

**Table 1:** Description of the Global Fund combination HIV prevention intervention for Adolescent Girls and Young Women aged 10–24 years from 2016 to 2019

Name	Description
KGIS^a^ (14–18 years)	A school-based programme which provided comprehensive SRH education, referrals to HIV testing and TB screening, linkage to care for HIV, pregnancy and other conditions, career guidance and homework support to encourage school attendance and completion of high school
SBC^b^ (10–14 years)	An in-school primary school programme for children struggling academically, affected by HIV or with signs of neglect. Components included linking and referring young people to health and other services, SRH education and peer support, promoting access to grants and an environment for ongoing learning and social cohesion.
RISE Clubs in- and out-of-school (15–24 years) or WOW^c^ Clubs (19–24 years) **(Cape Town only)**	Aimed to empower and build the resilience of young women and link them to biomedical services such as HTS, prevention of mother-to-child transmission, antiretroviral therapy, modern contraceptives and other SRH services, economic strengthening and SRH education.

^a^ *KGIS* Keeping Girls in School programme; *SRH* Sexual and Reproductive Health; *SBC* Soul Buddyz Clubs; *WOW*Women of Worth (WOW) clubs; *HTS* HIV Testing Services

^b^ SBC targeted boys and girls (10–14-years-old) and therefore could not include those currently in the survey, however the young girls may have been previously exposed to the club

^c^ Only participants in Cape Town had the opportunity to participate in the WOW Programme, and this question was not asked of participants in other districts

### Study design

The HERStory study used a mixed method design making use of quantitative and qualitative research methods to evaluate the combination HIV prevention intervention. This study presents findings from the quantitative survey related to intervention participation rates. It also presents qualitative data on AGYW intervention recipients’ perspectives of being recruited into and participating in the intervention, and intervention implementers’ and facilitators’ perspectives of recruiting AGYW into the intervention. The COREQ guidelines were followed when reporting on the methods used (Additional file 1).

### Sampling & Procedure

In South Africa there are nine provinces containing 52 districts; 10 highest HIV burden districts were selected for the AGYW intervention that is the subject of this study. These districts formed the sampling frame. The sampling for the quantitative and qualitative study components were independent of each other.

### Quantitative study

The survey was implemented in 6 of the 10 districts in which the intervention was implemented. These six districts (province) included: City of Cape Town (Western Cape), Ehlanzeni (Mpumalanga), OR Tambo (Eastern Cape), Tshwane (Gauteng), King Cetshwayo (KwaZulu-Natal), and Zululand (KwaZulu-Natal). The intervention implementers mapped the sub-areas in which the intervention was being implemented. A random sample of the 2011 available census small area layers (SALs) was then taken within the sub-areas in each district. We then conducted a systematic random sample of 35% of households within each SAL. Thereafter, all AGYW aged 15–24 years living in these selected households were invited to participate in the survey. A total of 4399 AGYW were enrolled into the household survey which started in September 2017 and was completed by November 2018. (For more information on the details of the sampling please see the HERStory report [17]).

The household survey was conducted in a private space, inside or outside the household, depending on where the participant felt comfortable. The survey was conducted on an electronic device (tablet) in the preferred language of the participant (English, isiXhosa, isiZulu, Setswana, siSwati) with the assistance of a fieldworker. The intervention implementers assisted the research team in identifying the languages spoken by participants living in the districts in which the intervention and evaluation took place. The fieldworker administered the survey; however, sensitive questions such as sex, HIV and violence were read by the fieldworker from the tablet and then the tablet was given to the participant to complete privately.

### Qualitative study

The qualitative study, conducted between August 2018 and March 2019, consisted of various methods including single one-time in-depth interviews (IDIs) as well as longitudinal serial individual interviews (SIDIs), and focus-group discussions (FGDs) with AGYW intervention beneficiaries. This study purposively sampled five of the ten districts in which the combination prevention intervention was implemented, to ensure that we included one district per implementing organisation. There were 5 implementing organisations consisting of NGOs and government institutions. The five districts (province) included: City of Cape Town (Western Cape); King Cetshwayo (KwaZulu-Natal); Gert Sibande (Mpumalanga); Bojanala (North West); and Nelson Mandela Bay (Eastern Cape). The districts were semi-urban and rural except for one district that was urban. Schools were purposively selected (two in each district) from a list of schools in which the intervention was implemented provided by the implementing organizations. The Life Orientation teachers helped us identify the learners who were in the intervention as well as their availability/ability of learners to attend the IDIs and FGDs. One out-of-school RISE club within each district was also selected from the list provided by the implementing organizations. With the assistance of the intervention implementers, a team of researchers invited the AGYW who had participated in the intervention to participate in the qualitative study. Members of the intervention implementing organisations were also invited to participate in the study. Participants were approached either face-to-face or over telephone or email.

Qualitative data collection took place in a venue which was private with no one other than the researchers and the participants present and where the participants felt safe. These venues were either at schools or in communities arranged through liaising with school staff and/or intervention implementers. All IDIs and FGDs, were conducted by female researchers trained and experienced in qualitative research methods, the study protocol, design, research tools, and human subject research ethics. KJ (PhD) and KM (Honors) conducted IDIs and FGDs with the AGYW intervention recipients, while ZD (PhD), JD (PhD) and CM (PhD) conducted the IDIs and FGDs with intervention implementers and facilitators. KJ, ZD, JD and CM are scientists and KM is a research technologist. They did not have a prior relationship with the participants and therefore were able to have a fairly unbiased stance during the conduction of the data collection. Researchers conducting the IDIs and FGDS were not part of the intervention study and were instead evaluating participants’ perspectives thereof and therefore did not bias the findings by trying to get participants to only give positive reports on the intervention. Researchers explained their positions very clearly to the participants as evaluators of the intervention and encouraged participants to be open and honest in their responses by asking them questions about what they think worked and did not work (please see Additional file 2 for more information on the topic guides). They introduced themselves to the participants and the focus of the research study before commencing with data collection. Individual Interviews were conducted predominantly face-to-face with a few serial interviews being done via telephone in the preferred language of the participants (English, isiXhosa, isiZulu, Setswana, siSwati) and were tape-recorded with the participant’s permission. FGDs were conducted face-to-face with participants speaking in their preferred language (English, isiXhosa, isiZulu, Setswana, siSwati). The intervention implementers assisted the research team in identifying the languages spoken by participants living in the districts in which the intervention and evaluation took place. The research team then ensured that those conducting the interview could speak the language of the participants in the district where data collection took place. FGDs consisted of 6–10 AGYW. During each IDI and FGD, there was a note taker present and a de-briefing report was completed after each IDI and FGD. Individual Interviews took between 20 and 40 min, and FGDs were between 40 and 90 min in duration. Data collection was concluded once data saturation was reached.

### Measures

#### Quantitative study

A composite indicator of AGYW’s participation was created in the intervention components. AGYW were defined as having participated in the intervention if they said yes to:

- having participated in a Soul Buddyz Club,
- participated in or been a member of a RISE Club,
- participated in or ever received a “cash incentive” for the Women of Worth programme (Cape Town participants only),
- attended a Keeping Girls at School health education session,
- or attended a homework support programme in the past year (The questionnaire developed for the quantitative HERStory study can be found in Additional file 3).

Biological measures included HIV testing. The HIV status of AGYW were determined using blood samples that were analysed in a laboratory. The survey also included self-reported questions about HIV testing and HIV status. Factors associated with participation were described in the intervention, including age, HIV status, socioeconomic status, whether the AGYW was attending high school or not, whether the AGYW had ever been pregnant (among those who reported having had sex), received social support from parents, whether the AGYW had a boyfriend in the past year, and whether the AGYW had experienced any form of intimate partner violence (IPV) and/or sexual violence in the past year. (For more details on the description of the measures, please see [17]).

#### Qualitative study

IDIs and FGDs were semi-structured and followed topic guides which included open-ended questions and probes (Additional file 2). These questions were piloted before the commencement of data collection in one of the districts by conducting interviews with a small number of AGYW. A few questions were then amended based on the pilot findings. Questions and topics included in this analysis were (for AGYW intervention recipients): “We would like to hear your experiences and views on different parts of the programme, a) Which parts worked well for AGYW like you? Why?, b) What parts did NOT work well for AGYW like you? Why?, c) What would you like to be added to these programmes so that they prepare AGYW to be strong, healthy and successful?”; (for intervention implementers and facilitators): “We would specifically like to hear about the process of setting up the projects: a) What worked well?, b) What challenges did you face? c) What would you do differently?”. Data triangulation was achieved by making use of a variety of sources in addition to interview transcripts. These sources included interviewer observations, fieldworker notes taken by a research assistant present during the interviews, post-interview debriefing reports which were conducted on a weekly basis with our research team to discuss thoughts and ideas emerging from the data, as well as follow-up ‘member checking’ analysis workshops with participants to review and discuss the preliminary findings and ensure data was interpreted accurately and clarify any misunderstandings. A brief demographic questionnaire was also administered to participants to gain information about their socio-demographic characteristics. The responses are presented further down in in the Results section under Table 2.

### Data analysis

Quantitative data was weighted due to the complex sampling design and survey-based analyses were completed using STATA/SE 14.2 [18]. All percentages reported are weighted. Descriptive summary statistics were used to describe the characteristics of participants by intervention participation status, stratified by age group. For these bivariate analyses, a Pearson Chi-square test was used, corrected for the survey-based analysis to describe whether HIV status, socioeconomic status, current enrolment in school, ever being pregnant, social support from parents, boyfriend in the past 12 months and IPV and/or sexual violence experience in the past 12 months were associated with participation in the intervention. Complete case analysis was applied when dealing with missing observations for both descriptive and bivariate analyses.

All audio-recordings were transcribed verbatim in the original language of the IDI/FGD (English, isiXhosa, isiZulu, Setswana, siSwati) and quality checked by the interviewer, then translated into English and quality checked by the interviewer for accuracy. Thematic analysis was used to explore responses from AGYW exposed to the intervention, and responses from implementers and facilitators of the intervention about their perspectives of participating and implementing the intervention, what they felt worked and did not work.

Data were coded initially using Nvivo 12 software. Analysis, conducted by two independent researchers, involved a focused word search of English transcripts/translations for relevant words such as, “participation”, “recruitment”, “retention” “incentive”, “gift”. Thematic analysis was initially deductive by using a pre-determined codebook (Additional file 4). This codebook was based on initial research questions used in the topic guides (Additional file 2) and then refined inductively from themes that emerged from the data. The data were then sorted into the different levels of influence. The two researchers compared notes and discussed the emergent themes via email and telephone on a weekly basis during the analysis process. A third researcher reviewed the themes and selected illustrative quotations to identify any aspects that were unclear or missing. Finally, all researchers discussed and agreed on themes and relevant quotes. Any discrepancies in interpretation were discussed until a consensus was reached. Feedback workshops were conducted with participants where the interviewer summarized the findings from the IDIs and FGDs and participants were given a chance to comment or clarify any misinterpretations.

## Results

*Demographic characteristics of the quantitative sample (*Table 3*):* Of the 4399 AGYW who completed the household survey, 56.7% were between the ages of 15 and 19 years old and the remaining 43.3% were 20–24 years of age. Almost half (48.4%) of AGYW living in the districts where the intervention was implemented, indicated that they had participated in at least one of the intervention components. Study laboratory tests found 12.4% of participants testing HIV positive of which 60.8% reported to have already known their status. Among AGYW who were HIV positive, almost 4.5% of them were recently infected. More than half of AGYW (56.2%) reported that they were in school at the time the survey was conducted. More than half (53.2%) of AGYW who had ever had sex (*N* = 3009) reported that they had ever been pregnant. Most AGYW (61.9%) reported that they had high social support from their parents. The majority of AGYW (67.3%) reported they had a boyfriend in the past 12 months. Almost a third (29.6%) of survey participants reported they had experienced IPV and/or sexual violence in the past 12 months.

**Table 2:** Demographic characteristics of AGYW in the qualitative sample who participated in the intervention

Variable	Frequency (%)
Age Group	
*15–18 years*	147 (79.5%)
*19–24 years*	38 (20.5%)
Languages^a^	
*IsiZulu*	40 (21.6%)
*Sesotho*	1 (0.5%)
*IsiXhosa*	75 (40.5%)
*Setswana*	16 (8.6%)
*Siswati*	29 (15.7%)
*English*	18 (9.7%)
*Afrikaans*	16 (8.6%)
*Tshivenda*	1 (0.5%)
Ever pregnant	
*Yes*	31 (16.8%)
*No*	154 (83.2%)
*Prefer not to say*	1 (0.5%)

^a^Participants could choose more than one language they commonly spoke

**Table 3:** Demographic characteristics of quantitative sample

Variable	Frequency (%^b^)
Age Group	
*15–19 years*	2515 (56.7%)
*20–24 years*	1884 (43.3%)
Participated in the intervention	
*Yes*	2103 (48.4%)
*No*	2296 (51.6%)
HIV Status	
*Positive*	568 (12.4%)
*Negative*	3829 (87.6%)
*Missing*	2 (0.03%)
Currently in school	
*Yes*	2518 (56.2%)
*No*	1881 (43.8%)
Ever pregnant^a^	
*Yes*	1642 (53.2%)
*No*	1332 (45.6%)
*Prefer not to say*	35 (1.2%)
Social support from parents	
*Low*	462 (10.3%)
*Moderate*	1207 (27.8%)
*High*	2730 (61.9%)
Had a boyfriend in the past 12 months	
*Yes*	2953 (67.3%)
*No*	1387 (31.4%)
*Prefer not to say*	59 (1.3%)
Experienced IPV and/or sexual violence in the past 12 months	
*Yes*	1263 (29.6%)
*No*	3136 (70.4%)

^a^ Only asked for those who reported to have ever had sex

^b^All percentages were weighted. Numerators were not weighted

### Factors associated with participation in the combination HIV prevention intervention

Table 4 presents the participation rates and the associated factors, stratified by age group. Among AGYW in both age groups combined, fewer participants who were HIV positive (43.3%; as per study laboratory tests), reported they had participated in at least one of the intervention components compared with those who were HIV negative (49.1%; *p* value = 0.001). The age-stratified results do not show an association between HIV status and participation in the intervention. No associations were found between AGYW’s socioeconomic status and their participation in the intervention. In both age groups, a greater proportion of AGYW who were in school at the time of the survey participated in the intervention compared with those who were not in school. Among both age groups, fewer AGYW who had ever been pregnant had participated in at least one of the intervention components compared with those who were never pregnant. No associations were found regarding social support from parents and participation rates. A greater portion of AGYW in both age groups who reported to have had a boyfriend in the past 12 months participated in the interventions compared with those who did not have a boyfriend in the past 12 months. Those aged 20–24-years-old who had experienced IPV and/or sexual violence in the past 12 months reported higher participation rates (43.2%) than those who did not experience IPV and/or sexual violence (38.8%, *p* value = 0.034), while no associations were found among 15–19-year-olds.

**Table 4:** Factors associated with participation in the combination HIV-prevention intervention) as measured by a household survey (weighted percentages)

Variable	Intervention Participation																				
	15–19-year-olds ***n*** = 2515							20–24-year-olds ***n*** = 1884							Total ***N*** = 4399						
	Yes			No			*P value	Yes			No			*P value	Yes			No			*P value
	n	%	95%CI	n	%	95%CI		n	%	95%CI	n	%	95%CI		n	%	95%CI	n	%	95%CI	
**HIV status**																					
Positive	98	55.0	49.0–60.8	87	45.0	39.2–51.0	0.849	136	38.0	33.4–42.8	247	62.0	57.2–66.6	0.226	234	43.3	39.7–46.9	334	56.8	53.1–60.3	0.001
Negative	1265	54.4	52.5–56.3	1064	45.6	43.7–47.6		602	41.0	38.7–43.3	898	59.0	56.7–61.3		1867	49.1	47.5–50.6	1962	50.9	49.4–52.5	
**Socioeconomic Status**																					
Higher	234	54.1	50.1–58.0	181	45.9	42.0–49.9	0.841	141	37.9	33.7–42.3	236	62.1	57.7–66.3	0.144	375	46.2	43.3–49.2	417	53.8	50.8–56.7	0.095
Lower	1130	54.5	52.5–56.5	970	45.5	43.5–47.5		598	41.2	39.0–43.5	909	58.8	56.5–61.1		1728	48.9	47.3–50.6	1879	51.1	49.4–52.7	
**Currently in school**																					
Yes	1203	58.5	56.4–60.5	871	41.5	39.5–43.6	< 0.001	252	58.4	54.4–62.3	192	41.6	37.7–45.6	< 0.001	1455	58.5	56.6–60.3	1063	41.5	39.7–43.4	< 0.001
No	161	36.3	32.6–40.1	280	63.7	59.9–67.4		487	35.2	33.0–37.4	953	64.8	62.6–67.0		648	35.4	33.6–37.4	1233	64.6	62.6–66.4	
Ever pregnant**																					
Yes	214	46.5	42.6–50.5	252	53.5	49.5–57.4	< 0.001	430	38.5	35.7–41.4	746	61.5	58.6–64.3	0.020	644	40.7	38.4–43.1	998	59.3	56.9–61.6	< 0.001
No	486	58.3	55.2–61.3	333	41.7	38.7–44.8		231	44.7	41.2–48.3	282	55.3	51.7–58.8		717	53.1	50.7–55.4	615	46.9	44.6–49.3	
**Social support from parents**																					
High support	847	54.4	52.1–56.7	709	45.6	43.3–47.9	0.211	471	41.5	38.9–44.2	703	58.5	55.8–61.1	0.207	1318	48.8	47.0–50.6	1412	51.2	49.4–53.1	0.116
Moderate support	371	53.1	50.0–56.1	335	46.9	43.8–50.0		183	37.7	33.8–41.7	318	62.3	58.3–66.2		554	46.6	44.1–49.0	653	53.4	51.0–55.9	
Low support	146	58.4	53.4–63.3	107	41.6	36.7–46.6		85	41.3	36.2–46.6	124	58.7	53.4–63.8		231	50.8	47.2–54.4	231	49.2	45.6–52.9	
Had a boyfriend in the past 12 months***																					
Yes	866	56.6	54.3–58.8	658	43.4	41.2–45.7	< 0.001	564	40.8	38.5–43.2	865	59.2	56.8–61.5	0.040	1430	48.9	47.2–50.7	1523	51.1	49.4–52.8	0.001
No	474	50.4	47.6–53.2	482	49.6	46.8–52.4		160	38.4	34.4–42.5	271	61.6	57.5–65.6		634	46.5	44.2–48.9	753	53.5	51.2–55.8	
**Experienced IPV and/or sexual violence in the past 12 months**																					
Yes	335	56.0	52.5–56.3	248	44.0	40.7–47.4	0.273	297	43.2	40.3–46.2	383	56.8	53.8–59.7	0.034	632	49.1	46.8–51.4	631	50.9	48.6–53.2	0.430
No	1029	53.9	51.8–56.0	903	46.1	44.0–48.2		442	38.8	35.9–41.8	762	61.2	58.2–64.1		1471	48.1	46.3–49.8	1665	51.9	50.2–53.7	

*P value for the difference between those who participated in the intervention and factors associated with participation

** Only asked for those who reported to have ever had sex

***n = 49 “prefer not to answer” responses excluded

****n = 59 “prefer not to answer” responses excluded

A total of 185 AGYW who were recipients of the intervention were interviewed and included in focus group discussions (as seen in Table 5 below). One AGYW intervention recipient refusal was received due to the parental consent being declined. Nineteen of the 185 AGYW participated in SIDIs and 2 were lost to follow up when researchers were unable to contact them. In-depth interviews and FGDs were also conducted with 13 intervention implementers and 13 intervention facilitators.

**Table 5:** Qualitative study sample and data collection details

Sample Group^a^	Total	City of Cape Town, Western Cape		King Cetshwayo, KwaZulu-Natal		Gert Sibande, Mpumalanga		Bojanala, North West		Nelson Mandela Bay, Eastern Cape	
		IDI (SIDI)	FGD	IDI (SIDI)	FGD	IDI (SIDI)	FGD	IDI (SIDI)	FGD	IDI (SIDI)	FGD
**Intervention recipient AGYW 15–24 years** (*N* = 185)	57 IDI (18 SIDI), 19 FGD	5 (2)	5	17 (5)	3	10 (4)	5	16 (5)	2	9 (3)	4
**Intervention Implementers** (*N* = 13)	13 IDI	2	0	2	0	4	0	3	0	2	0
**Intervention Facilitators** (N = 13)	11 IDI, 2 FGD	3	0	2	1	0	1	2	0	4	0

^a^ The AGYW who participated in the qualitative study were not the same AGYW as those who participated in the quantitative study

^b^IDI: In-depth interview; SIDI: serial individual interviews; FGD: Focus group discussion

*Demographic characteristics of the AGYW who participated in the intervention qualitative sample:* The median age of AGYW living in the communities in which the intervention was implemented was 17 years (Interquartile range (IQR) 15–24). The most commonly spoken language was isiXhosa (40.5%), followed by isiZulu (21.6%).

### Participation in the intervention: facilitators and barriers reported by AGYW and intervention implementers and facilitators

Themes which emerged from the qualitative data were coded and grouped into facilitators and barriers to participation which were encountered by AGYW, intervention implementers and facilitators of the combination HIV prevention intervention. The facilitators to participation included motivation towards participation in the interventions. Barriers included misguided expectations about financial rewards or job opportunities; competing responsibilities, interests or activities; family responsibilities including childcare; inappropriate incentives; inability to disrupt the school curriculum and difficulties with conducting interventions after school hours due to safety concerns; miscommunication about meetings; as well as struggles to reach out-of-school AGYW. These findings were then grouped into the following levels of influence which are presented below: 1) Individual; 2) Interpersonal; 3) Organisational; and 4) Community.

### Individual level

#### Motivation

A key facilitator for AGYW’s recruitment into intervention components related to motivation. At the individual level, participation was enabled when AGYW felt motivated to attend. AGYW respondents expressed self-motivation driven by curiosity and an active interest in extra-curricular activities at school.

*I love exploring things. If there is something happening at school, I go and find out what is happening, that is how I joined RISE. (AGYW 15-18yrs, Eastern Cape)*

Altruism, community mindedness, and the desire to help other AGYW in their communities were also motivating factors for attendance.

*We felt that since we are young girls in our community…we have children and there is more to… learn from RISE. So that we can also help other kids… So that’s how we started. Then we asked for information on how RISE works, how do we attend, and what do you do in your meetings so we can attend. (AGYW 19-24yrs, North West)*

*Even with RISE, nobody ever told me to go and join or how it will help me, I just joined it on my own… because I am a person who especially loves to work with other youth people. (AGYW 19-24yrs, KwaZulu-Natal)*

Perceived benefits of the intervention also provided motivation. Some AGYW joined the intervention because they were struggling with peer pressure and they perceived that they might get support to cope through the intervention.

*I joined RISE due to peer pressure because I had many friends and they were involved in drugs, and I learned from RISE about how to deal with substances and pressure and I decided to stop going to friends and associated myself with those who were not on drugs. (AGYW 15-18yrs, Eastern Cape)*

Perceived benefits also included the fun activities organised for participants, for when trips or outings were planned, some AGYW were motivated to attend on those days:

*Not all of us were attending, because others didn’t come, they were not serious…(they) will only come if we are going somewhere out of (place name), that’s when they will come…they just want a ride obviously, they want to see places (AGYW 19-24yrs, North West)*

Intervention facilitators also reported that they thought the certificates AGYW were given after completion of the sessions acted as a motivator for participants:

*Another thing that attracts them is because we award them with certificates after finishing the sessions… So, we tell them that if you were bunking (also known as skipping school) and have not attended 16 session or plus, you don’t get the certificate, so I think it is the main thing that attracts them maybe to finally come to the sessions. (Intervention Facilitator, KwaZulu-Natal)*

Individual level motivation was shown to be important not only for AGYW intervention recipients, but also for intervention facilitators. When participants did not attend the sessions, intervention facilitators reported they felt demotivated because they had already spent money traveling to the venue. However, they weighed their disappointment against the potential benefits of reaching even one or two participants.

*Sometimes, honestly speaking sometimes when I am supposed to secure appointments with RISE I feel that, you know what, maybe I must not go…but, at the same time, the two that come makes a difference…That’s what I always say… at least they tried and whatever they need to discuss you can still discuss it. I am not going to say because the other eighteen did not come, I won’t support these kids. But it demoralizes that one comes from (place name) going to, for example, (place name). I pay R8.00 (approximately US$ 0.46), for transport instead of using the money for bread…And they do not come, at all! (Intervention Facilitator, North West)*

### Incentives

Material incentives were used as an approach to keep young women motivated to attend and participate in the intervention components. AGYW in KwaZulu-Natal reported that they were given sanitary towels, umbrellas, and water bottles when they participated in the KGIS programme. Facilitators in North West reported giving mugs, glasses, and selfie sticks after 3 months of attendance in the intervention. WOW members in the Western Cape received a cash incentive, R300 (approximately US$ 17.37) for recruiting 15 participants. Some AGYW reported that they valued the incentives.

Some participants felt that the opportunity to learn and be part of a group was sufficient motivation to attend, and that the provision of incentives and refreshments was an added bonus.

*(They) only (gave us) knowledge, and to get us away from thinking bad things, to gather together. (AGYW 19-24yrs, KwaZulu-Natal)*

Some intervention facilitators reported that incentives facilitated participation in the intervention.

*What we found very difficult… People who are not working, when you call them they expect you to give them something for their time… They are not going to just give you their time for free, that’s where the headache was with RISE out-of-school…. then we found that there is money for refreshments so we used that as the bribe as per session…. it worked… It kept them coming and then we had incentives as well. (Intervention Facilitator, Eastern Cape)*

*Money is a challenge, cause when we don’t have money to buy them snacks they don’t come. (Intervention Facilitator, Eastern Cape)*

However, in some instances even when incentives were provided, AGYW were insufficiently motivated to attend.

*The RISE Club… it’s a cool idea, it just doesn’t take off in every school. We also then offer incentives for the club attendees. So if you attend 3 sessions… either we will give you cup or umbrella, or earphones, so they get a little incentive at the end of the 3 sessions… also… you get say R200 (approximately US$ 11.58) per session and then at the end some of them accumulate it, they can buy something to eat or they can do their own stationery for the project... (but) the club idea never takes off. (Western Cape, Intervention Implementer)*

The intervention implementers and facilitators felt that some of the gifts provided to AGYW participants were inappropriate, or even counter-productive. For example, providing school going learners with earphones as an incentive, was viewed as potentially detrimental to their education, as they would be distracted in class.

*Most of our incentives are not good for the children. Just like earphones, you can’t give earphones to the school kids because she needs to listen towards what the teacher are saying. (Intervention Implementer, KwaZulu-Natal)*

Other incentives that were felt to be inappropriate included selfie-sticks and glass mugs. More useful and contextually relevant incentives would have been items such as toiletries, or vouchers to enable AGYW to purchase necessary toiletries.

*We do give them incentives but that’s not what they need. We give them airtime, we give them water bottles but that’s not something that they need because they can take that bottle and throw it away. They need something like roll on, sprays, toiletries something that they can use. (Intervention Facilitator, Eastern Cape)*

*Like for instance for incentives we had earphones… We had… selfie stick… I don’t see why do you need that thing, your phone has selfie mode, so why need a stick I don’t know… I wish we could have incentives that make sense…Why can’t you give me a Spar voucher for R100 (approximately US$ 5.79), where I can go and buy myself toiletries? (Intervention Facilitator, Eastern Cape)*

Suggestions were made by AGYW themselves about what incentives would make them more likely to attend the interventions, such as organising outings, or providing programme branded t-shirts so that they can be identified by others and also promote the intervention to others:

*Most girls are not eager to come and attend, they need nice things like going out on trips. (AGYW 15-18yrs, Mpumalanga)*

*We want things that are visible like maybe getting a t-shirt and show it. When someone sees it, I will then explain about how I got it from this group. (AGYW 15-18yrs, Mpumalanga)*

The availability of intervention participants to attend sessions was an individual-level barrier that was cited for retention. Intervention implementers and facilitators explained that retaining AGYW in the intervention proved to be challenging for reasons including time constraints and lack of availability, and AGYW’s competing responsibilities, interests, or activities, which made it difficult for AGYW to attend meetings consistently:

*There’s been poor attendance… I’m losing hope because people have other commitments, such as going to school, jobs, others have boyfriends… So for me to get a hold of them… It’s getting tougher, but at the end of the month… I do end up finding them. (Intervention facilitator, North West)*

*Sometimes the girls are completing other things or maybe they just needed to eat lunch or something. So then attendance dropped, that’s the challenge, the biggest challenge of RISE is that they get recruited they start up with a bang and they will start up with 25 in a class and by the end of the quarter they down to 8 or 9 or 10. (Intervention implementers, Cape Town)*

### Misguided expectations

It seemed that AGYW had a misguided expectation that the intervention would provide them with what they felt was their most important needs, such as employment opportunities or money, but were later disillusioned when they found they would not receive them, and this led them to no longer be motivated to attend.

*When I joined RISE, the moment you hear woman empowerment you thought like that… like maybe it does more maybe they will take us as woman, they will give us small jobs maybe we cut trees and then they pay us that small money. (AGYW 19-24yrs, North West)*

The intervention implementers reiterated this saying that some of these young women have children to support and come from child headed household and need money to put food on the table and therefore desire quick fixes to their economic difficulties. Participants wanted to know how they would benefit from attending the clubs, specifically if they would be paid for attendance:

*The hype is always high, everyone wants to be part of the club right… sometimes most of them they get disappointed that they are not giving them a daily job so they start falling off and going because the dynamic and the challenges they are faced with…Some they stay here because they can see the bigger picture, there’s just going to be a struggle it going to take long but at the end of the day there’s light at the end of the tunnel. These are the girls that are having children, they need to feed them these are the girls that are child headed families, they need to put bread on the table. So they want something that is quick quick that can give them money. (Intervention implementer, North West)*

The provision of incentives and payment was a particularly important motivator for attendance that took place in the “out-of-school” clubs. Intervention facilitators found recruiting outside of the school more challenging than in schools.

*In school they are bound by being within the school grounds and they can’t leave, while with the out-of-school if they don’t feel like attending, they won’t (Intervention facilitator, KZN)*

### Interpersonal level

#### Good facilitators

Factors at the interpersonal level which AGYW felt facilitated participation and attendance included having good facilitators that the AGYW felt they could be open with:

*When we joined RISE Club we did not open up completely because we were under the impression that she (the facilitator) is an adult and will not understand what we were going through and we limited ourselves. She told us to open up because while we were at RISE Club we are all the same and experiencing the same things including her so we must talk and learn to be confident in speaking. (AGYW 15-18yrs, Eastern Cape)*

Intervention implementers and facilitators described interpersonal barriers to participation among AGYW who are in the older age group (19–24 years), as many had conflicting obligations, limiting their availability to dedicate time to attend intervention activities. Even those AGYW who they wanted to attend, often experienced challenges related to childcare and parenting responsibilities.

*If you have got a big number of people with children and on the day that you decide to do you RISE Out-of-school Clubs they won't have a baby sitter but also they can't afford one…They are not gonna come, if they sacrifice 2 weeks or 3 weeks over a period of their time knowing that at the end they will have outcomes they do that… (Intervention implementer, Eastern Cape)*

*(She) will have an issue about the baby, or she should go and collect her sister, such excuses things like that... (Intervention facilitator, KwaZulu-Natal)*

Also described were instanced of resistance from family members towards the AGYW participating in interventions.

*You find that a child is interested but the parent won’t allow her to attend (Intervention facilitator, KwaZulu-Natal)*

Additionally, some participants were prevented from attending due to resistance from boyfriends.

*Some girls could not come to the training and when we ask around what happened you will get answers like the boyfriend did not want them to attend… (Intervention implementer, North West)*

### Organisational level

On the organisational level, it proved to be challenging for intervention implementers to target specific school-going AGYW of certain ages because ages and grades do not always correlate and learners are therefore not all the same age in each grade, making intervention activities hard to implement. Intervention implementers suggested it would be better to invite the whole grade regardless of their ages because all the girls in their community are at risk.

*(Schools would say) “The mere fact that we are based in this community in this area all of our girls are at risk so we can’t hand pick individuals from classes, we going to give you the whole grade 9”. So, in none of our school did we have the hand-picked. Everybody gave us the whole class, the whole grade. (Intervention implementer, Western Cape)*

Safety was also a concern in some schools and learners needed to be transported to and from school. The intervention implementers and facilitators were then unable to have classes after school hours and had to conduct them during interval (recess), but this became a problem because some would want to eat lunch and if the intervention did not feed them then that would discourage them from coming:

*The biggest challenge was safety in our communities the kids are living in high risk communities so after they get transported in and out-of-school and if you after school programmes they miss their transports, so that’s why it shifted to interval but if you are then taking interval away and not giving them something to eat... (Intervention implementer, Cape Town)*

### Communication

Furthermore, there were challenges with communication reported by existing members, who said they were not informed about the meetings, and then subsequently reprimanded for their absence.

*Some of us you don’t inform us, and then afterwards you say we don’t attend meetings, and then we get shocked. I just heard you now complaining that we don’t want to do it. You didn’t even tell me about it, or to show that there is a project. There was a certain meeting where you were just reprimanding me. That like, “you never show up for meetings. You only show up when (facilitator’s name) comes.” Whereas for some of us when there is a meeting, we don’t get told. (AGYW 15-18yrs, North West)*

Communication about school-based intervention activities was often provided to learners at morning assembly, which means that those AGYW who arrived at school late were not aware of the activities being offered.

*Most of the learners, they are clueless about what is happening here at school, because they are coming late at school, where most of the things are announce in assembly, and that affect(s) them. (AGYW 15-18yrs, Western Cape)*

Suggestions made by AGYW to resolve communication challenges included providing intervention participants with a few days warning, and regular reminders.

### Community level

On the community level, intervention facilitators expressed that recruitment for out-of-school AGYW was more challenging than recruiting AGYW in schools, some went door-to-door themselves and others appointed others to recruit for them.

*In the community, I recruited children myself, I had to go door-to-door looking for these kids, if I happen to know that, a particular house has a child that has dropped out-of-school…that I know is within this age group. That is how I recruited out-of-school. (Intervention facilitators, KwaZulu-Natal)*

Intervention implementers adapted their recruitment methods to be responsive to specific communities.

Suggestions for improving recruitment were also made by AGYW, such as holding events in the community to attract and promote the interventions which will give participants an opportunity to ask questions about the intervention.

*We know that like young woman we love having fun, when you pass by and hear sound of music from the hall, obviously you will come because you want to see what’s happening and then when you enter you will maybe see the (organisation) flag, posters from (the organisation), and then you will seek more information about what (the organisation) is actually doing… (AGYW 19-24yrs, North West)*

*Maybe it can do, I don’t know an event or something…there’s a RISE event, this is what happens maybe at the hospital or clinic, they’ll come with their own gazebos and setup for testing…“there’s a RISE event over there, they’re even testing”, and so on, you see. (AGYW 19-24yrs, North West)*

Intervention facilitators did however say that they had awareness campaigns in the communities to promote the interventions where a participant who completed the course would come and speak about the benefits of the intervention:

*You find that we do awareness campaigns, a child who has been attending the programme that has just been finished, goes and speaks to other kids, and it shows that those kids have changed from what she was (Intervention facilitator, KwaZulu-Natal)*

## Discussion

This study sought to investigate the factors associated with AGYW’s participation in a combination prevention intervention and their perspectives of participation, as well as the perceptions of intervention implementers and facilitators relating to what worked and what did not work. Our quantitative results reveal that almost half of AGYW (48.4%) living in the selected districts in which the intervention was implemented participated in at least one of the intervention components. This is a high level of intervention coverage and shows that the intervention was reaching the population for which it was designed.

In both age groups, this study found that AGYW in school were more likely to have participated in at least one intervention component compared with those no longer in school with substantial absolute differences in participation rates (> 20%) between those in school and those not in school. Our findings suggest those who were HIV positive or had ever been pregnant, who might have needed the intervention most, were less likely to have been reached than those who were HIV negative or had never been pregnant. Nevertheless, the differences in coverage were small, and even among adolescents who were HIV positive or who had ever been pregnant, approximately one third to one half had participated in at least one intervention component. This research shows that AGYW who had a boyfriend in the past 12 months, who might be more vulnerable to HIV acquisition or transmission, were more likely to have participated in at least one intervention component, compared to those who had not had a boyfriend in the past 12 months. In the 20–24 year age group, AGYW who had experienced IPV and/or sexual violence in the past 12 months, who were also more likely to be vulnerable to HIV, were more likely to have been reached than those who had not experienced IPV and/or sexual violence in the past 12 months. Nonetheless, the levels of participation were relatively high across these subgroups.

The qualitative findings corroborate the quantitative findings that reaching AGYW in schools was more successful than reaching those out of school. This is further confirmed by statements from a review on out-of-school youth which state that most interventions have targeted in-school youth which are easier to reach and make interventions cheaper and less complex, since school attendance is mandatory [19]. Furthermore, participants trust the school administration and participants are therefore likely to want to participate if the study is done through the school [19]. However, reaching those in school was not without challenges. Structural barriers were met such as the inability to disrupt the school curriculum and to conduct interventions after school hours due to safety concerns. Therefore, facilitators’ only option was to make use of interval sessions which limit the time they were able to run sessions. In an after-school SRH intervention conducted in the Western Cape, South Africa among youth aged 13 years it was found that structural barriers related to transport and competing responsibilities were key to reasons for non-attendance [16]. Those who may benefit most from the after-school interventions might be least likely to attend due to these barriers. Mathews et al. [16] suggest that arranging safe transport for participants home would have helped them overcome many barriers to attendance of after-school interventions.

In our qualitative findings, the challenge expressed by intervention implementers in reaching those who have already left school speaks to the quantitative findings of out-of-school AGYW who were less likely to have been reached. A review conducted among out-of-school youth in Eastern and Southern Africa speaks about the importance of SRH interventions to include those hard to reach out-of-school youth who were found to be associated with risky sexual behaviour [19]. In another HIV combination prevention intervention in Zambia and South Africa, they found engaging the community from the start of an intervention was important when conducting interventions within the community setting [20]. While community engagement was initiated from the start of our intervention, perhaps improvements could have been made on how to better engage the potential participants. Incentives such as money, refreshments, and gift vouchers have also been shown to play an important role in improving participation in out-of-school interventions [21].

Our qualitative data show that incentives were a key facilitator in motivating AGYW to participate in the intervention. Previous studies also emphasize that compensation can be important when including participants from economically disadvantaged areas [22]. A previous study on retention strategies and factors associated with missed visits in an HIV prevention trial found that women viewed monetary compensation as a benefit of study participation and that it should be reflective of the time and risk associated with participation [23]. The compensation amount for their study varied by site and ranged between $25 and $50s for face-to-face visits and between $10 and $15 for updates done over the phone. However, another study among African American teens (aged 13–17 years) which looked at enhancing recruitment and retention in intervention studies, reported that although participants thought that money is important to attract adolescents into an intervention study, food and fun were more important in order to retain them [24]. AGYW in our study reported that some intervention components did not offer incentives; however, they were appreciative of the refreshments received. It appears that receiving food is seen as an incentive for participation in these resource-limited or economically disadvantaged areas. Further research is needed to assess whether this is true for participants living in areas of low socio-economic status. Intervention implementers and facilitators in our study however felt that the interventions that did provide incentives, often provided items that were inappropriately selected for AGYW, and that contextually relevant incentives were needed such as toiletries or vouchers. This indicates that intervention implementers and facilitators were not part of the decision making when it came to decide on which incentives to be given to participants. Formative research involving intended beneficiaries in the design of the interventions is needed for future programme designing. A needs assessment, focused on issues such as food insecurity or lack of hygiene supplies, would determine what incentives would be most beneficial and ethical in assisting with recruitment.

The qualitative findings support the quantitative findings that AGYW who had ever been pregnant were less likely to participate. Among the individual level barriers identified in the qualitative analysis, AGYW in this study faced barriers in participating in intervention components due to childcare responsibilities and their lack of access to childcare services. Barriers such as these have also been reported by previous intervention studies, with participants finding it difficult to find time to spend away from their family to attend interventions, having other commitments, as well as challenges with transport to get to the intervention site [25–27]. In order to reduce this barrier, other intervention studies provided informal childcare for participants which included toys and snacks for children [23].

Our qualitative findings revealed other facilitators and barriers that affect participation in the intervention which were not explored in the quantitative research. These facilitators have been classified into the levels of influence. On the individual level, a key facilitator to participation was motivation. Motivation was reported in all forms, AGYW expressing self-motivation where they volunteered to join the activities either due to being driven by curiosity and an interest in extra-curricular activities at school; their desire to want to help other communities, also known as altruism; through seeking help with peer pressure or wanting to be part of the fun activities planned by the interventions. Individual motivation was important not only for participants but for intervention facilitators. Some intervention facilitators expressed feeling demotivated when participants did not attend.

Additionally, there appeared to be a misguided expectation among AGYW that there would be financial rewards and job opportunities for attendance, which later lead to dropout (attrition) when discovering this was untrue. In reports from a pilot Randomized Controlled Trial (RCT) community based intervention conducted in the United States, it was found that “not fully understanding the study requirements and incentives was one of the key factors leading to a high dropout rate” [28]. Researchers therefore conducted interviews with participants to develop an easy-to-read flyer that outlined the study’s purpose, expectations and incentives to reduce the risk of a high dropout rate. It is possible that AGYW participants, who mostly came from resource-constrained communities were primarily motivated by the financial benefits of participation.

On the interpersonal level, AGYW spoke about facilitators to intervention participation, such as provision of session/workshop facilitators that participants felt open to speak with. Having good facilitators speaks to the importance of having facilitators who are adolescent friendly when working with AGYW on sensitive topics such as SRH. By adolescent friendly, we mean, by not criticizing adolescents even if you do not approve of their words and actions, reaching out to them in a friendly manner and treating them with empathy and sensitivity [29]. This will allow them to be open and honest during intervention participation. These factors motivate AGYW to stay in the intervention and improve retention rates. Other studies substantiate this finding with evidence on the impact of health workers negative attitudes’ which prevent adolescents from being open with them about their problems and seeking health care [8]. Another study conducted in South Africa also experienced the challenge of enrolling and retaining AGYW who were controlled by their partners or parents who are not in support of their participation in HIV prevention interventions [25]. Research in this area state that participants value the opinion of their family members and friends and would not agree to participate in studies if their loved ones are not happy with them participating [30]. Community buy-in is therefore important in order to combat these interpersonal level barriers, by making participants and their partners aware of the interventions being conducted in their communities and the benefits of participation. One would have thought that AGYW who were survivors of IPV and/or sexual violence would be more likely to be discouraged to attend by their abusive partners; however, we could not confirm this with our quantitative analysis, which proved the opposite, that survivors of abusive partners were more likely to attend. It is encouraging that survivors of violent relationships are being reached.

Organisational level challenges expressed by intervention implementers that AGYW spoke about was the need to improve communications when it comes to dates and times for meetings as school announcements in the morning which can be missed by those who come late to school. Some suggestions from AGYW were to remind them a day or two before the meeting. It is also evident as proven by other studies, that sending reminders to participants as well as using multiple methods of contact (mail, email, in person, text messaging) play a significant role in achieving a high retention rate [27, 31, 32].

Recommendations on ways in which participation could be improved were made by study respondents. Suggestions included having events and awareness campaigns to attract participants where the current members can show case their talents and entertain people from the community and promote the intervention by sharing the benefits and their personal experiences of the interventions while wearing branded clothing. AGYW also mentioned that activities such as outings organised by the interventions were motivators for AGYW to attend. The importance in having adolescent friendly intervention facilitators whom AGYW feel they can be open with during studies on sensitive matters such as SRH was expressed by AGYW, this might have helped with retention, illustrating the importance of having good facilitators. Other intervention studies also found that among the factors that contribute to attendance was when trust was developed between the participants and the study staff conducting the recruitment [20, 32]. To the knowledge of the authors, there are no studies that were conducted in South Africa related to participation in a combination HIV and pregnancy prevention and management interventions such as this one.

### Limitations

AGYW who participated in the qualitative study were not the same as those who participated in the quantitative study. AGYW who indicated in the survey that they had ever been pregnant, might not have carried through with their pregnancies and kept their babies. We also need to acknowledge, that not all AGYW in our sample may have been “in need” of HIV and pregnancy prevention or management interventions. The lessons learned from this study may be specific to this study population, context, and intervention type and therefore care should be taken when attempting to generalize these findings to other populations, contexts, or interventions.

## Conclusion

Effective coverage of an intervention can only be attained when taking into consideration the factors associated with participation in interventions, as well as the facilitators and barriers influencing participation. This study identified facilitators and barriers reported by AGYW, intervention implementers and facilitators to recruitment into and attendance of components of a combination HIV prevention intervention. Recruiting AGYW into SRH interventions is challenging and presents with many barriers as demonstrated by the findings of this study. Addressing some of the findings mentioned in this study can lead to improvements. Designers of combination HIV prevention interventions need to address these structural barriers to participation so that AGYW can attend without having to risk their safety and compromising their family, childcare and schooling responsibilities. Strategies to create demand need to include clear communication about the nature and potential benefits of such interventions, and the inclusion of incentives which will be valued by participants. Furthermore, innovative ways to motivate out-of-school youth to participate in interventions are needed. To the best of our knowledge, there has not previously been any study of this kind in South Africa. These findings can help implementers to plan future interventions or improve and adjust current interventions.

## Supplementary Information

**Additional file 1.** COREQ template. Consolidated criteria for reporting qualitative studies (COREQ): 32-item checklist

**Additional file 2.** Qualitative topic guide. Interview and focus group discussion guides for AGYW (intervention recipients) and programme implementers

**Additional file 3.** HERStory Survey for YWG aged 15–24 (English). The full questionnaire for the quantitative HERStory study for AGYW aged 15–24 years.

**Additional file 4.** HERStory Qualitative Study Codebook. Pre-determined codebook used for analysis of qualitative data

## Data Availability

The quantitative dataset analysed during the report of the current study are available on the HERStory website, https://www.samrc.ac.za/intramural-research-units/HealthSystems-HERStory. The qualitative study data report is undergoing the funders approval and will be available when ready from the corresponding author on reasonable request.

## Abbreviations

AGYW: Adolescent girls and young women
EC: Effective coverage
FGDs: Focus group discussions
HTS: HIV testing services
IDIs: In-depth interviews
IPV: Intimate partner violence
KGIS: Keeping girls in school
RCT: Randomized controlled trial
SALs: Small area layers
SBC: Soul buddyz clubs
SIDI: Serial Individual Interviews
SRH: Sexual and reproductive health
STIs: Sexually transmitted infections
UHC: Universal health coverage
WOW: Women of worth
