# Supplementary material for: Factors influencing adolescent girls and young women’s participation in a combination HIV prevention intervention in South Africa
Source: BMC Public Health. 2021 Feb 27;21:417. doi: 10.1186/s12889-021-10462-z (PMC7912506; doi:10.1186/s12889-021-10462-z)
Supplement: Supplementary file 2 — Additional file 2. Qualitative topic guide. Interview and focus group discussion guides for AGYW (intervention recipients) and programme implementers [file 12889_2021_10462_MOESM2_ESM.pdf]

Project Title: Impact Evaluation of the Global Fund Young Women and Girls (YW&G) Intervention in Ten South African Districts

QUALITATIVE GUIDE:

INTERVIEWS AND FOCUS GROUP DISCUSSIONS WITH YOUNG WOMEN AND GIRLS 15-24 YEARS – INTERVENTION RECIPIENTS

Version 3: 11th October 2018

Introductory Script for Interviewer:

Thank you for agreeing to be part of this study and to take part in an interview or group discussion. Our goal is to hear the stories and experiences of young women and girls.

There are programmes that have been running in your school that provide support to female learners by providing peer education, health education, homework support, career guidance, as well as counselling, education and health services. Some schools have also been offering clubs that offer life skills and empowerment activities, and link young women to educational and economic opportunities and health services. Some of these programmes are run in the community around the school.

1. What do you know about programmes like these offered in your school?
    1. What do you know about programmes like these offered in your community?

2. Tell me about your experiences been with these programmes?
    - an experience related to the programme that you enjoyed the most?
    - an experience related to the programme that you enjoyed the least?

3. We are interested in hearing your stories about how the programme has affected your life.
    - How has the programme, or things you learned in the programme changed:
        - your daily life? – examples? *(Personal stories)*
        - how you feel about yourself?
        - how you act in your romantic relationships?
            - relationships you are in now
            - relationships you might have in the future
        - how you plan for the future?

4. We would like to hear your experiences and views on different parts of *the programme*

a) Which parts did you think worked well for young women and girls like you? Why?

b) What parts of the club do you think do NOT work well for young women and girls like you? Why?

c) What would you like to be added to these programmes so that they prepare young women and girls to be strong, healthy, and successful?

5. To what extent have you received any support to help change your educational experience?
    - To what extent did things you learned help you to do better in school? - examples
    - To what extent did things you learned change the way you face future challenges in your education? – examples
6. Have there been any other differences in your life or the lives of your family and community that you want to share with us?

<u>Health System Experiences</u>

We are interested in hearing from you about the health education and health services you and your friends receive at school and in the community. We are especially interested in health education and health services that focus on HIV, TB, and other STIs, teen pregnancy, and stress and depression, and your experiences with these.

7. What **health education** have/do you and your friends receive/d at school?

8. What **health services** have/do you and your friends receive/d at school?

9. To what extent do these services meet you and your friends' needs?
    a. What could be improved about these services?
    b. What are the best parts of getting services for:
        - HIV testing? /HIV treatment? /TB? /Sexual and reproductive health (STIs, Pregnancy, Contraceptives)? /Mental health?
    c. What is the most challenging part?

<u>Individual and Family Experiences</u>

10. We are interested in hearing about how young women and girls like you have happy and safe sexual relationships.
    - Can you describe
        - your ideal romantic partner?

- the way your partner/s treat you in your romantic and sexual relationships?

11. We would like to hear about how young women and girls like you look after their health in relationships and about what you need to support you to protect yourself and your health in relationships.
    a) How much do you think about health and well-being your daily life?
    b) How much do you worry about your health?
    c) What makes it easy or difficult for young women and girls like you to use condoms?
    d) What makes it easy to ask a partner about their HIV status?
    e) To what extent do you worry about violence in your relationships?
12. What adults in your life are most helpful in helping you meet the challenges of being a young women or girl in South Africa? (Probe for parents, family, teachers, health workers)
    - What kinds of support have they given you?
    - If you have not received all the support you need from adults in your life, what kind of support do you wish you had received? And from whom?

School Experiences and Future Planning

We would like to hear about your school life.

13. Could you tell us about any things that make it difficult to attend school and achieve good grades?

14. What kind of support do you get at school to help you:
    - attend school and do well?
    - make plans for your future?

15. What has helped you most to prepare for your future?

Closing

We want to make sure that we have gathered all the advice that we can from young women and girls like you.

16. In this community, what things do you think would help improve the lives of young women and girls like you?

Project Title: Impact Evaluation of the Global Fund Young Women and Girls (YW&G) Intervention in Ten South African Districts

QUALITATIVE GUIDE:
INTERVIEWS WITH PROGRAMME IMPLEMENTERS

Global Fund Principal Recipients and Sub Recipients

Version 3
22nd August 2018

Introductory Script for Interviewer:
Thank you for agreeing to be part of this study and to take part in an interview or group discussion. Our goal is to hear the views and opinions of Programme Implementers for the Global Fund Programmes.

We would like to hear about your experiences implementing the Global Fund projects.

1. As an introduction, could you tell us:
   - Which programme you have been involved with?
   - Which community it has been implemented in?
   - What is your role in the implementation?
   - How long have you been involved in this project?

2. How would you describe your experiences implementing the following:
   - ***Soul Buddyz Clubs***
   - ***Rise Clubs***
   - ***Keeping Girls in School Programme***
   - *Other components of the intervention*

3. We would specifically like to hear about the process of setting up the projects
   - What worked well
   - What challenges did you face
   - What would you do differently?
   - Were you supported in this process?
   - What would have improved this?

4. We would like to hear about your relationships with existing structures in the communities
   - To what extent did you link up to existing structures?
     - Describe and give details
   - What worked well?
   - What was challenging?
   - What would you differently?
   - Were you supported in this process?
   - What would have improved this?

   - To what extent did the intervention strengthen **the school system**?
     - How and in what way?
     - Probe on:
       - Care provided

        - Data systems
        - Capacity building

- To what extent did the intervention strengthen **the health system**?
    - How and in what way?
    - Probe on:
        - Care provided
        - Data systems
        - Capacity building

- To what extent did implementation activities aim to generate ownership of the intervention by:
    - schools
    - communities
    - How and in what way?

- To what extent was the intervention integrated into:
    - the health system
    - education system
    - What features influenced the extent to which it was integrated?
    - Probe on:
        - Intervention alignment with priorities of health and education systems
        - Intervention physically integrated into health system, financially and operationally integrated.
    - To what extent did the integration result in the intervention being seen to be "co-owned?

- To what extent would you say that there were power issues between implementers and key role players?
    - In schools
    - In the health system?
    - To what extent were the relationships between implementers and schools/communities based on trust?

- To what extent did the organisational culture of existing structures affect implementation?
    - Schools
    - Health services

5. We would like to hear about your relationships with other implementing partners
    - To what extent did you collaborate with other implementing partners?
        - Describe and give details
    - What worked well?
    - What was challenging?
    - What would you differently?
    - Were you supported in this process?
    - What would have improved this?

    - In what ways could you have established the relational aspects of the partnerships differently?

- To what extent did you have, or not have enough time for establishing the relational aspects of the partnerships?

6. We would like to hear about your relationships and linkages with other services
    - To what extent did you have relationships and linkages with other services?
        - Describe and give details
    - What worked well?
    - What was challenging?
    - What would you differently?
    - Were you supported in this process?
    - What would have improved this?

7. How would you describe the impact that these programmes have had on the communities they have been implemented in?
    - Describe & explain
    - Positive impact
    - Negative impact

8. What advice would you give to someone / organisations setting up similar projects in the future?
    - How should one manage the implementation of external interventions in ways to maximise implementation?
    - How should one manage the implementation of external interventions in ways to maximise sustainability?

9. To what extent were there mid-stream adaptions to the intervention activities in response to:
    - Contextual constraints
    - Iterative learning
        - What were these adaptions?
    - To what extent did shocks and stressors in the wider context affect implementation?
        - Probe on violence

<u>Closing</u>

We want to make sure that we have gathered all the advice that you may have for us.

10. In this community, what things do you think would help improve the lives of young women and girls?
    1. What could help young people to:
        - stay healthy and happy?
        - feel loved and accepted?

We really value your opinions and we would like to thank you for your time. To end our discussion, is there any other advice you would like to give us or any other information you want to share with us?
