# Supplementary material for: Factors influencing adolescent girls and young women’s participation in a combination HIV prevention intervention in South Africa
Source: BMC Public Health. 2021 Feb 27;21:417. doi: 10.1186/s12889-021-10462-z (PMC7912506; doi:10.1186/s12889-021-10462-z)
Supplement: Supplementary file 3 — Additional file 3. HERStory Survey for YWG aged 15–24 (English). The full questionnaire for the quantitative HERStory study for AGYW aged 15–24 years. [file 12889_2021_10462_MOESM3_ESM.pdf]

HERStory Survey for YWG aged 15-24 (English)

Last Modified by: Elsa Marshall **on** 25 Oct 2018 15:55:17 **Revision number:** 1896 **Field Count:** 457

Section 1. Variables

1.1 test

Text

This field is not displayed on the handset, Value: {{test}}

1.2 hhid

Text

This field is not displayed on the handset, Value: {{hhid}}

1.3 province_Adolescent

Numeric

This field is not displayed on the handset, Value: {{province}}

1.4 district_Adolescent

Text

This field is not displayed on the handset, Value: {{district}}

1.5 subdistrict

Text

This field is not displayed on the handset, Value: {{subdistrict}}

1.6 sal

Text

This field is not displayed on the handset, Value: {{sal}}

1.7 mapnumber

Text

This field is not displayed on the handset, Value: {{mapnumber}}

1.8 gpslat

Text

This field is not displayed on the handset, Value: {{gpslat}}

1.9 gpslong

Text

This field is not displayed on the handset, Value: {{gpslong}}

1.10 caregiver_id

Text

This field is not displayed on the handset, Value: {{caregiver_id}}

1.11 adolescent_id

Text

This field is not displayed on the handset, Value: {{adolescent_id}}

1.12 household_id

Text

This field is not displayed on the handset, Value: {{household_id}}

1.13 intro1

GLOBAL FUND IMPACT EVALUATION SURVEY FOR YOUNG WOMAN AND GIRLS (OVER 18 YEARS OLD) SELECT 'NEXT' TO BEGIN Select NEXT to continue

Expects a single option response (**required**), Default: NEXT

- [ ] NEXT [0]

1.14 GPS

Capture the GPS co-ordinates. [Click 'Options' at the bottom of the screen and choose 'Get Location']

Expects a latitude and longitude coordinate (optional)

1.15 date_today

What is today's date?

Expects a date response (**required**)

Constraints

*Response must be **Less Than or Equal'DATENOW'***

1.16 minor

Is the participant a minor? (Someone under the age of 18)

Expects a single option response (**required**)

- [ ] Yes [1]
- [ ] No [0]

Branches

If response **Equals 'No [0]'** then skip to ***conf_eli (1.18)***

1.17 inclusion_criterea

Please select any that apply...

Expects multiple selected options (**required**)

- [ ] Female aged 15-24 years [1]
- [ ] Lives in the household [2]
- [ ] If under 18 years of age, parent, guardian, caregiver or household representative has consented [3]
- [ ] Willing to provide written informed consent and to participate in the study [4]
- [ ] None of the Above [5]

1.18 conf_eli

Please note that the participant is not eligible any of the following applies:

Expects a single option response (**required**), Default: None of the above , the participant is eligible

- [ ] Under 18 years of age and parent/guardian/foster parent/caregiver unwilling to give consent [1]
- [ ] Unwilling to participate [2]
- [ ] Cognitive or mental challenges (based on the assessment of the participant's ability to comprehend the study information provided) [3]
- [ ] Deaf or mute. [4]
- [ ] Unable to speak English, IsiZulu, isiXhosa, Northern Sotho, Sotho, Tswana, Tsonga, Swazi, Swati, Sepedi, Afrikaans [5]
- [ ] Not available for participation between 8 a.m. and 9 p.m [6]
- [ ] None of the above , the participant is eligible [7]

Branches

If response **Equals 'None of the above , the participant is eligible [7]'** then skip to ***bar_1 (1.20)***

1.19 Not eligible

Based on the selected criteria, the participant is not eligible, please select Go back to change your response, and check that the participant is eligible to participate. Select End survey now if participant is not eligible for this study.

Expects a single option response (**required**)

- Go back [0]
- End survey now. [1]

Branches

If response **Equals 'Go back [0]'** then skip to ***conf_eli (1.18)***

If response **Equals 'End survey now. [1]'** then skip to ***end (23.1)***

1.20 bar_1

Please scan participants's Barcode/QR code. Scan participant SAMPLE barcode [Note to Fieldworker: Barcode is the partcipant's study ID, which links to lab results]. Select Options > Scan QR Code in order to scan the barcode assigned to this participant

Expects a numeric response (**required**)

1.21 Participant barcode re enter

Please RESCAN the participant SAMPLE bar code. Select Options > Scan QR Code in order to scan the barcode

Expects a numeric response (**required**)

Constraints

*Response must be **Equals'q984376'***

1.22 cell_number

Please enter your Cell phone number so that we can remind you via SMS to visit your nearest clinic and collect your results. *If you do not have a Cell number ,please provide someone in this household's number who we could SMS to inform you to go collect your results. Please note that no results will be sent via SMS , this is only a reminder for you to collect your results!

Expects a phone number (**required**)

Section Prerequisites
Skip when *minor (1.16)* Equals'Yes [1]'

Section 2. Consent forms 18+

2.1 instruct_consent

INFORMED CONSENT FOR ENROLLMENT 18 TO 24 YEARS OLD *Note to Fieldworker* Please read out paper-based consent form for ENROLLMENT to the participant and ask them to sign at the bottom of the page in the space provided , as well as sign the statement on the tablet. Select NEXT for the participants statement and signature *Please remember to give a the signed copy of the consent form to the participant*

2.2 participant_statement

I, have had all of the above Information explained and I understand the explanation. I have been given answers to my questions about the procedures involved in the study. I have received a copy of the information sheet regarding enrollment . I AGREE to ...

Expects multiple selected options (**required**)

- ☐ Participate in this study and I have received a copy of the information sheet [0]
- ☐ Complete the questionnaire [1]
- ☐ Provide a blood sample [2]
- ☐ None of the above [3]

2.3 permission_db_link

I AGREE to have my data linked to government databases in the future.

Expects a single option response (**required**)

- ☐ Yes - I agree [1]
- ☐ No - I refuse. [0]

2.4 agree_recontact

I AGREE to be contacted by the study team to participate in future studies.

Expects a single option response (**required**)

- ☐ Yes - I agree to be re-contacted. [1]
- ☐ No - I refuse to be re-contacted [0]

2.5 part_sign_1

Participant Signature: Select options and select signature capture Fieldworker, please guide the participant to use their finger or a stylus to draw their signature onto the screen. Do not turn the tablet to the side.

Expects an image response (**required**)

2.6 HCT_offer

I have been offered a rapid HIV test using the finger prick method. If I accept this test, I will get my HIV test results today, within 15 minutes of the finger prick.

Expects a single option response (**required**)

- ☐ I understand that I will be able to have an HIV test today and receive my results, if I so choose [1]

2.7 cons_sample

INFORMED CONSENT FOR SAMPLE STORAGE 18 TO 24 YEARS OLD *Note to Fieldworker* Please read out paper-based consent form for SAMPLE STORAGE to the participant and ask them to sign at the bottom of the page in the space provided , as well as sign the statement on the tablet. Select NEXT for the participants statement and signature *Please remember to give a the signed copy of the consent form to the participant*

2.8 agree_storage_participate

I, have had all of the above Information explained and I understand the explanation. I have been given answers to my questions about the procedures involved in the study. I AGREE to participate in this study. I have received a copy of the information sheet regarding sample storage.

Expects a single option response (**required**)

- ☐ I agree that my blood sample can be stored for future study. [1]
- ☐ I do not want my blood sample to be stored for future study [2]

2.9 sign_storage

Participant Signature: Select options and select signature capture Fieldworker, please guide the participant to use their finger or a stylus to draw their signature onto the screen. Do not turn the tablet to the side

Expects an image response (**required**)

2.10 FW_statement

I (Fieldworkers name and surname) declare that I have explained the information given a signed copy of the informed consent for enrollment AND Sample storage for 18 to 24 years. He/She was encouraged and given ample time to ask me questions. Our conversation was conducted in English, IsiZulu, isiXhosa, Northern Sotho, Sotho, Tswana, Tsonga, Swazi, Swati, Sepedi, Afrikaansi and no translator was used. Was a copy of the signed copy given to the participant?

Expects a single option response (**required**)

- Agreed,our conversation was conducted in English [1]
- Agreed, our conversation was conducted in Afrikaans [2]
- Agreed, our conversation was conducted in Xhosa [3]
- Agreed, our conversation was conducted in Sesotho [4]
- Agreed, our conversation was conducted in Isizulu [5]
- Agreed, our conversation was conducted in Swati [6]
- Disagree [0]

2.11 fw_sign

Fieldworkers's signature: Select options and select signature capture: Fieldworker you will now sign your name: Please use your finger or a stylus to draw your signature onto the screen. Do not turn the tablet sideways.

Expects an image response (**required**)

Section Prerequisites
Skip when *minor (1.16)* Equals'No [0]'

Section 3. Assent forms Under 18

3.1 instruct_consent_15_17

INFORMED ASSENT FOR ENROLLMENT 15 TO 17 YEARS OLDS *Note to Fieldworker* Please read out paper-based consent form for ENROLLMENT to the participant and get them to sign at the bottom of the page in the space provided , as well as sign the statement on the tablet. Select NEXT for the participants statement and signature *Please remember to give a the signed copy of the consent form to the participant*

3.2 participant_statement_2

I, have had all of the above Information explained and I understand the explanation. I have been given answers to my questions about the procedures involved in the study. I have received a copy of the information sheet regarding enrollment . I AGREE to ...

Expects multiple selected options (**required**)

- Participate in this study and I have received a copy of the information sheet [0]
- Complete the questionnaire [1]
- Provide a blood sample [2]
- None of the above [3]

3.3 permission_db_link2

I AGREE to have my data linked to government databases in the future.

Expects a single option response (**required**)

- Yes - I agree [1]
- No - I refuse. [0]

3.4 agree_recontact2

I AGREE to be contacted by the study team to participate in future studies.

Expects a single option response (**required**)

- Yes - I agree to be re-contacted. [1]
- No - I refuse to be re-contacted [0]

3.5 part_sign_1_2

Participant Signature: Select options and select signature capture Fieldworker, please guide the participant to use their finger or a stylus to draw their signature onto the screen. Do not turn the tablet to the side.

Expects an image response (**required**)

3.6 HCT_offer_2

I have been offered a rapid HIV test using the finger prick method. If I accept this test, I will get my HIV test results today, within 15 minutes of the finger prick.

Expects a single option response (**required**)

- I understand that I will be able to have an HIV test today and receive my results, if I so choose [1]

3.7 cons_sample_2

INFORMED ASSENT FOR SAMPLE STORAGE 15 TO 17 YEARS OLD *Note to Fieldworker* Please read out paper-based consent form for SAMPLE STORAGE to the participant and get them to sign at the bottom of the page in the space provided , as well as sign the statement on the tablet. Select NEXT for the participants statement and signature *Please remember to give a the signed copy of the consent form to the participant*

3.8 agree_storage_participate_2

I, have had all of the above Information explained and I understand the explanation. I have been given answers to my questions about the procedures involved in the study. I AGREE to participate in this study. I have received a copy of the information sheet regarding sample storage.

Expects a single option response (**required**)

- I agree that my blood sample can be stored for future study. [1]
- I do not want my blood sample to be stored for future study [2]

3.9 sign_storage_2

Participant Signature: Select options and select signature capture Fieldworker, please guide the participant to use their finger or a stylus to draw their signature onto the screen. Do not turn the tablet to the side

Expects an image response (**required**)

3.10 FW_statement_2

I (Fieldworkers name and surname) declare that I have explained the information given a signed copy of the informed assent for enrollment AND Sample storage. He/She was encouraged and given ample time to ask me questions. Our conversation was conducted in English, IsiZulu, isiXhosa, Northern Sotho, Sotho, Tswana, Tsonga, Swazi, Swati, Sepedi, Afrikaans and no translator was used. Was a copy of the signed copy given to the participant?

Expects a single option response (**required**)

- Agreed, our conversation was done in English [1]
- Agreed, our conversation was done in Afrikaans [2]
- Agreed, our conversation was done in Xhosa [3]
- Agreed, our conversation was done in Sesotho [4]
- Agreed, our conversation was done in Isizulu [5]
- Agreed, our conversation was done in Swati [6]
- Disagree [0]

3.11 fw_sign_2

Fieldworkers's signature: Select options and select signature capture: Fieldworker you will now sign your name: Please use your finger or a stylus to draw your signature onto the screen. Do not turn the tablet sideways.

Expects an image response (**required**)

Section 4. Introduction

4.1 Inst1

Good day my friend! Thank you for taking time out today and joining us on the the HERStory study. We are part of a research team from Epicentre and the South African Medical Research Council. What will you be helping me with today? Well, today you will be completing this very important form with me. Why do I need this information from you? Your story is important. We want to learn from YOU! The information I will get from you today will help me know more about young people like yourself in South Africa. Each section you complete will help us understand the successes and challenges of young people in South Africa. YOU have the power to influence the solutions we find for young people in South Africa. How does this form work? There are various sections- each with specific questions on your life and health. All you need to do is select the option by touching the screen [FIELDWORKER to demonstrate how to use tablet] Select NEXT to continue

Expects a single option response (**required**), Default: NEXT

- NEXT [0]

4.2 Inst2

Remember this is not a test and there is no right or wrong answer! We want you to be honest with us. If you are uncomfortable with sharing any information with me that is also fine- you can just select I do not want to answer option . If you want a break, just tell the person on our team. If any questions upset you, you can stop at any time. You do not have to give a reason. Your decision to answer questions or stop being in the study is entirely up to you. Will anyone know about the important information you share with me? The information you give me will not be shared with any other participants, friends, parent or guardian or caregiver, teachers or nurses. I really value the information you give the Epicentre team and I. We will keep it a secret. No one will know the information is yours as this form will not contain your name, just a unique project number. The only time we will not be able to keep it a secret is if you tell us you plan to harm someone, or you tell us that you plan to harm yourself, or if you tell us the name of someone who is abusing you. If you tell us these things, we are required by law to report them so that we can arrange help for you, or for the person you plan to harm. Select NEXT to continue

Expects a single option response (**required**), Default: NEXT

- NEXT [0]

4.3 Inst3

What do you do if you are unsure about any of my questions? If you are unclear about any big words that I use, please ask me and I will explain them to you. For example, if you don't know what a caregiver is, I will tell you it is someone who acts like a parent to you and is responsible for you. For example, they might give you love, take care of you when you are ill, make your food, and iron and washes your clothes. For the questions that are very private, I will let you enter your answers in the device in a way that I cant see your answers. I will read the question and then give you the device for you to enter your answer privately. I will not be able to see your answer after you have entered it. When it comes to these private questions, you will need to know how to answer. Here is some information.

Expects a single option response (**required**), Default: NEXT

- NEXT [0]

4.4 Inst4

What do you do if none of the options listed match your answer? For certain questions, some of the options may not contain your specific answer, please feel free to select Other (please specify), once selecting this option , it will take you to a text box where you can add in your answer. What do you do if you have more than one answer? Some questions will have an instruction telling you (You may choose more than one option) this will allow you to select all the options that match your answer What do you do if you are unsure how to operate the Tablet ? When you are answering the private questions, if you are unsure how the device works or if you need assistance with answering then please ask me. I will help you without seeing your answer OK, are we ready? Cool, lets go!

Expects a single option response (**required**), Default: Begin Survey

- Begin Survey [0]

Section 5. Demographics

5.1 Demo_intro

DEMOGRAPHICS First, I would like to get to know you by asking you a few questions about you and your life.

5.2 self_pride

What are you most proud of about yourself?

Expects a long text response (**required**)

5.3 DOB

What is your date of birth (dd-mm-yyyy)?

Expects a date response (**required**), Default: 01-01-1900

Constraints

*Response must be **Greater Than or Equal'01/01/1900'** AND*

*Response must be **Less Than or Equal'01/01/2007'***

5.4 CalculateAge

Operator

This field is not displayed on the handset, Operator: Set(**AgeDays (5.5)** , DAYSBETWEEN(Q(984407) , DATENO)))

5.5 AgeDays

Numeric

This field is not displayed on the handset

5.6 partAgeYears

Operator

This field is not displayed on the handset, Operator: Set(**AgeYears (5.7)** , ROUND(SUB(Division of (Q(984409) ,365.25) ,0.5) ,0))

5.7 AgeYears

Numeric

This field is not displayed on the handset

5.8 AgeConfirmation

Please confirm that your CURRENT age is Q(**AgeYears (5.7)**) years old. If this is incorrect, Select NO so that we can adjust your date of Birth

Expects a single option response (**required**)

- Yes [1]
- No-go back and change Date of birth [2]

Branches

If response **Equals 'No-go back and change Date of birth [2]'** then skip to ***DOB (5.3)***

5.9 age

My CURRENT Age is Q(**AgeYears (5.7)**) Press Next at the bottom of the screen to continue

Expects a numeric response (**required**), Default: Q(**AgeYears (5.7)**)

5.10 language

What is your home language?

Expects a single option response (**required**)

- Afrikaans [1]
- English [2]
- Ndebele [3]
- Northern Sotho [4]
- Sotho [5]
- Sepedi [6]
- Swati [7]
- Swazi [8]
- Tsonga [9]
- Tswana [10]
- Venda [11]
- IsiXhosa [12]
- IsiZulu [13]
- Other (Specify) [98]

Prerequisites
Skip when ***language (5.10)*** **Not Equal 'Other (Specify) [98]'**

5.11 other_language

If Other , please specify...

Expects a single line text response (**required**)

5.12 nationality

What is your nationality?

Expects a single option response (**required**)

- South African citizen [1]
- Non-citizen (Permanent resident) [2]
- Non-citizen (Refugee) [3]

5.13 race

How do you identify yourself?

Expects a single option response (**required**)

- African [1]
- White [2]
- Coloured [3]
- Indian/Asian [4]
- Other (Specify) [98]

Prerequisites
Skip when ***race (5.13)*** **Not Equal 'Other (Specify) [98]'**

5.14 other10_2

If Other, please specify...

Expects a single line text response (**required**)

5.15 community

How long have you lived in this community?

Expects a single option response (**required**)

- Always [1]
- 1-5 years [2]
- More than 5 years [3]

5.16 relationship status

What is your CURRENT relationship status?

Expects a single option response (**required**)

- Single (not in a relationship , no partner at all) [1]
- Dating someone (in a relationship, but not living together) [2]
- Dating and living together, not married (living with boyfriend/girlfriend/partner) [3]
- Married (not living with husband/wife) [5]
- Married (currently living with husband/wife) [6]
- Divorced / separated [7]
- Widowed [8]
- I prefer not to say [97]
- Other (specify) [98]

Prerequisites
Skip when ***relationship status (5.16)*** **Not Equal 'Other (specify) [98]'**

5.17 other_rel

If Other , Please specify...

Expects a single line text response (**required**)

5.18 rel HH

What is your relationship to the head of this household?

Expects a single option response (**required**)

- I am the head of the household [1]
- Daughter [2]
- Wife/partner [3]
- Granddaughter [4]
- Niece [5]
- Sister [6]
- Friend [7]
- Other (Specify) [98]

Prerequisites
Skip when ***rel HH (5.18)*** **Not Equal 'Other (Specify) [98]'**

5.19 other_relationship_hhh

If Other , please specify...

Expects a single line text response (**required**)

5.20 awayhome

In the past 12 months have you been away from your usual home for more than one month?

Expects a single option response (**required**)

- Yes [1]
- No [0]

5.21 nightsaway

In the past week, how many nights have you stayed away from home?

Expects a numeric response (**required**)

5.22 highestpassed

What is your highest grade COMPLETED? [NOT THE GRADE THE PARTICIPANT IS CURRENTLY IN]

Expects a single option response (**required**)

- Grade R / Pre-school [1]
- Sub a/class 1/grade 1 [2]
- Sub b/class 2/grade 2 [3]
- Standard 1/grade 3 /ABET 1 [4]
- Standard 2/grade 4 / ABET 2 [5]
- Standard 3/grade 5/ABET 2 [6]
- Standard 4/grade 6/ABET 3 [7]
- Standard 5/grade 7/ABET 3 [8]
- Standard 6/grade 8/ABET 3 [9]
- Standard 7/grade 9/ABET 3 [10]
- Standard 8/grade 10/NTC 1 [11]
- Standard 9/grade 11/NTC 2 [12]
- Standard 10/grade 12/NTC 3 [13]
- Further studies incomplete [14]
- Diploma/other post school completed [15]
- Further degree completed [16]
- Don’t know [17]
- No schooling [19]

5.23 inschool

Are you currently in school?

Expects a single option response (**required**)

- Yes [1]
- No [0]

Branches

If response **Equals 'No [0]'** then skip to ***leaveschool (5.41)***

5.24 currentgrade

What grade are you currently in?

Expects a single option response (**required**)

- Grade 1 [1]
- Grade 2 [2]
- Grade 3 [3]
- Grade 4 [4]
- Grade 5 [5]
- Grade 6 [6]
- Grade 7 [7]
- Grade 8 [8]
- Grade 9 [9]
- Grade 10 [10]
- Grade 11 [11]
- Grade 12 [12]

5.25 hwk

In the past year, have you attended a homework support programme?

Expects a single option response (**required**)

- Yes [1]
- No [0]

5.26 absentfreq

In the past year, how often were you absent from school?

Expects a single option response (**required**)

- I was never absent [1]
- I was rarely absent (1-5 days per year) [2]
- I was occasionally absent (1-3 days per month) [3]
- I was regularly absent (1-3 days per week) [4]
- I was absent for several weeks at a time [5]

5.27 absentmore1wk

In the past year, were you ever absent from school for more than a week?

Expects a single option response (**required**)

- Yes [1]
- No [0]

Branches

If response **Equals 'No [0]'** then skip to ***timegetschool (5.30)***

5.28 absentreason

In the past year, when you were absent from school for more than a week, why were you absent? (You may choose more than one option)

Expects multiple selected options (**required**)

- I have been sick [1]
- I don't feel safe going to school [2]
- I don't feel safe while in school [3]
- I don't like school [4]
- I have to look after my younger brothers/sisters/other children [5]
- I have to look after a sick family member [6]
- There's not enough money to send me to school everyday [7]
- school is too far away [8]
- I have to work [9]
- I missed school because of my period (menstruation) [10]
- Exams are done [11]
- I am pregnant/have a baby/have a child [12]
- Other (Specify) [98]

Prerequisites
Skip when ***absentreason (5.28)*** **Excludes 'Other (Specify) [98]'**

5.29 Other3

If Other , please specify

Expects a single line text response (**required**)

5.30 timegetschool

In the past year, how long did it take you to get to school most mornings?

Expects a single option response (**required**)

- Less than 30 minutes [1]
- 30 minutes to 1 hour [2]
- More than 1 hour [3]

5.31 schooltransport

In the past year, how did you get to school most mornings? (You may choose more than one option)

Expects multiple selected options (**required**)

- Walk [1]
- Bus [2]
- Taxi [3]
- Private car [4]
- Train [5]
- Bicycle [6]
- Motorbike [7]

5.32 costschool

In the past year, how much did it cost for you to get to and from school most days?

Expects a single option response (**required**)

- R0 [1]
- Less than R10 [2]
- R10-R20 [3]
- More than R20 [4]
- I don't know [97]

5.33 moneygoods

In the past year, have you or your family received money or goods (such as a uniform, food) from a programme to stay in school?

Expects a single option response (**required**)

- Yes [1]
- No [0]

Branches

If response **Equals 'No [0]'** then skip to ***loanbursary (5.37)***

5.34 institutionmoneygoods

Which institution/s provided the money or goods?

Expects a single line text response (**required**)

5.35 rec_money_stay_school

Have you or your family ever received money or goods (such as a uniform, food) from a programme to stay in school?

Expects a single option response (**required**)

- Yes [1]
- No [0]

Branches

If response **Equals 'No [0]'** then skip to ***loanbursary (5.37)***

5.36 inst_money_goods

Which institution/s provided money or goods (such as a uniform, food) from a programme to stay in school?

Expects a single line text response (**required**)

5.37 loanbursary

In the past year have you or your family received a loan?

Expects a single option response (**required**)

- Yes [1]
- No [0]

Branches

If response **Equals 'No [0]'** then skip to ***leaveschool (5.41)***

5.38 institutionloanbursary

Which institution/s provided the loan or bursary?

Expects a single line text response (**required**)

5.39 loan_school

Have you or your family ever received a loan or bursary (money lent or given to you) so that you can attend school?

Expects a single option response (**required**)

- Yes [1]
- No [0]

Branches

If response **Equals 'No [0]'** then skip to ***leaveschool (5.41)***

5.40 inst_loan_school

Which institution/s provided the loan so that you can attend school?

Expects a single line text response (**required**)

Prerequisites
Skip when ***inschool (5.23)*** **Equals 'Yes [1]'**

5.41 leaveschool

When did you leave school?

Expects a single line text response (**required**), Default: (Month/Year)

Prerequisites
Skip when ***inschool (5.23)*** **Equals 'Yes [1]'**

5.42 reasonleaveschool

Why did you leave school?

Expects a single option response (**required**)

- I completed Grade 12 [1]
- I was sick [2]
- I was bullied [3]
- I was pregnant [4]
- I needed a job [5]
- I needed to look after people at home [6]
- I was not learning much at school [7]
- My school did not have good teachers [8]
- I have special education needs and my school was not meeting my needs [9]
- Other (specify) [98]

Prerequisites
Skip when ***reasonleaveschool (5.42)*** **Not Equal 'Other (specify) [98]'**

5.43 Other4

If Other , please specify

Expects a single line text response (**required**)

Prerequisites
Skip when ***inschool (5.23)*** **Equals 'Yes [1]'**

5.44 currentlydoing

What are you currently doing? (You may choose more than one option)

Expects multiple selected options (**required**)

- Studying at college or University [1]
- Learning a trade [2]
- Starting/running a business [3]
- Looking for a job [4]
- Working [5]
- Taking care of my baby [6]
- Taking care of family members [7]
- Taking care of the household [8]
- Nothing [9]
- Other (specify) [98]

Prerequisites
Skip when ***currentlydoing (5.44)*** **Excludes 'Other (specify) [98]'**

5.45 other_doing

If Other, Please specify...

Expects a single line text response (**required**)

Prerequisites
Skip when ***inschool (5.23)*** **Equals 'Yes [1]'**

5.46 uni

Have you ever attended a college or university to study further after school?

Expects a single option response (**required**)

- Yes [1]
- No [0]

Branches

If response **Equals 'No [0]'** then skip to ***havemoney (5.50)***

Prerequisites
Skip when ***inschool (5.23)*** **Equals 'Yes [1]'**

5.47 currentuni

Are you currently attending a college or university?

Expects a single option response (**required**)

- Yes [1]
- No [0]

Branches

If response **Equals 'No [0]'** then skip to ***havemoney (5.50)***

Prerequisites
Skip when *inschool (5.23)* Equals 'Yes [1]'

5.48 yearsuni

How many years have you studied at college or university?

Expects a numeric response (optional)

Prerequisites
Skip when *inschool (5.23)* Equals 'Yes [1]'

5.49 helppayuni

In the past year, have you attended a career jamboree or career day or career expo at college or university??

Expects a single option response (**required**)

- Yes [1]
- No [0]

5.50 havemoney

I am now going to ask you questions about employment and earning money. Do you have your own money?

Expects a single option response (**required**)

- Yes [1]
- No [0]
- I prefer not to say [97]

5.51 bankacc

Do you have your own bank account?

Expects a single option response (**required**)

- Yes [1]
- No [0]
- I prefer not to say [97]

5.52 save

Do you save money? (For example do you put money aside for another day or a special purpose?)

Expects a single option response (**required**)

- Yes [1]
- No [0]
- I prefer not to say [97]

5.53 owemoney

Do you owe anyone money?

Expects a single option response (**required**)

- Yes [1]
- No [0]
- I prefer not to say [97]

5.54 career

In the past year, have you attended a career jamboree or career day or career expo at school?

Expects a single option response (**required**)

- Yes [1]
- No [0]

5.55 cv

In the past year, have you received help from an organisation in your community to write a CV?

Expects a single option response (**required**)

- Yes [1]
- No [0]

5.56 smallbusiness

In the past year, have you received help from an organization in your community to start a small business?

Expects a single option response (**required**)

- Yes [1]
- No [0]

5.57 interviewskills

In the past year, have you received training on job interview skills from an organisation in your community?

Expects a single option response (**required**)

- Yes [1]
- No [0]

5.58 findjob

In the past year, have you received help to find a job from an organisation in your community?

Expects a single option response (**required**)

- Yes [1]
- No [0]

5.59 oftenwork

In the past year how often did you work to earn money?

Expects a single option response (**required**)

- A few days a week [1]
- Each month [2]
- Most months [3]
- Once in a while [4]
- Never worked [5]

Prerequisites
Skip when ***oftenwork (5.59)*** **Equals 'Never worked [5]'**

5.60 dayswrk

In the last 4 weeks, how many days did you work for yourself or other people to earn money?

Expects a numeric response (optional)

5.61 wrkactivities

In the last 12 months have you done any of the the following: (You may choose more than one option)

Expects multiple selected options (**required**)

- Searched for work [1]
- Searched newspapers for jobs [2]
- Handed in or sent off an application for work [3]
- Worked WITHOUT pay to get experience [4]
- Worked WITH pay to get experience [5]
- Developed an idea for a way of earning by selling or making things [6]
- Asked for funding for a business plan [7]
- Earned money through selling or making things [8]
- None of the above [9]

Section Prerequisites
Skip when ***age (5.9)*** **Less Than'18'**

Section 6. House Hold Survey for YWG aged 18- 24

6.1 place_brth

I am now going to ask you a few questions about you and your family. Where were you born?

Expects a single option response (**required**)

- South Africa [1]
- Lesotho [2]
- Botswana [3]
- Namibia [4]
- Zimbabwe [5]
- Swaziland [6]
- Malawi [7]
- Mozambique [8]
- Nigeria [9]
- Democratic Republic of Congo (DRC) [10]
- Other (please specify) [98]

Prerequisites
Skip when ***place_brth (6.1)*** **Not Equal 'Other (please specify) [98]'**

6.2 other1

If Other , please specify...

Expects a single line text response (**required**)

6.3 identity_doc

Do you have a South African ID?

Expects a single option response (**required**)

- Yes [1]
- No [0]

Prerequisites
Skip when ***identity_doc (6.3)*** **Equals 'Yes [1]'**

6.4 no_id

Why don't you have a South African ID?

Expects multiple selected options (**required**)

- Cannot afford to pay for the ID [1]
- Cannot afford the transport to go to Home Affairs [2]
- Long waiting queues at home affairs [3]
- I was not in school when they were making IDs at school [4]
- Lack all the required documents (affidavit from young woman/girl’s parent or close relative, birth certificate, colour photographs) [5]
- No one to take care of other siblings whilst away [6]
- It is lost or was stolen [7]
- I am not a South African citizen [8]
- Other (Specify) [98]

Prerequisites
Skip when *no_id (6.4)* **Excludes 'Other (Specify) [98]'**

6.5 other2

If Other , please specify...

Expects a single line text response (**required**)

6.6 bio_mom

Is your biological mother (birth mother) alive?

Expects a single option response (**required**)

- Yes [1]
- No [0]
- I dont know [99]

Branches

If response **Equals 'I dont know [99]'** then skip to *bio_dad (6.12)*

If response **Equals 'No [0]'** then skip to *bio_dad (6.12)*

6.7 bio_mom_liv

Does your biological mother (birth mother) live here?

Expects a single option response (**required**)

- Yes [1]
- No [0]

Prerequisites
Skip when *bio_mom_liv (6.7)* **Equals 'Yes [1]'**

6.8 bio_mom_know

Do you know your biological mother (birth mother)?

Expects a single option response (**required**)

- Yes, I know her and I've met her [1]
- Yes, I know her but I have never met her [2]
- No, I don't know who she is [0]
- Other [98]

Prerequisites
Skip when *bio_mom_know (6.8)* **Not Equal 'Other [98]'**

6.9 other_know_biomom

Please specify:

Expects a single line text response (**required**)

6.10 bio_mom_rel

Is your biological mother (birth mother) legally or traditionally married?

Expects a single option response (**required**)

- Yes [1]
- No [0]
- I dont know [99]
- Other (Specify) [98]

Prerequisites
Skip when *bio_mom_rel (6.10)* **Not Equal 'Other (Specify) [98]'**

6.11 Other8_2

If Other , Please specify...

Expects a single line text response (**required**)

6.12 bio_dad

Is your biological father (birth father) alive?

Expects a single option response (**required**)

- Yes [1]
- No [0]
- I dont know [99]

Branches

If response **Equals 'No [0]'** then skip to ***rest_hh (6.18)***

If response **Equals 'I dont know [99]'** then skip to ***rest_hh (6.18)***

6.13 bio_dad_liv

Does your biological father (birth father) live here?

Expects a single option response (**required**)

- Yes [1]
- No [0]

Prerequisites
Skip when ***bio_dad_liv (6.13)*** **Equals 'Yes [1]'**

6.14 bio_dad_know

Do you know your biological father (birth father)?

Expects a single option response (**required**)

- Yes, I know him and I've met him [1]
- Yes, I know him but I have never met him [2]
- No, I don't know who he is [0]
- Other [99]

Prerequisites
Skip when ***bio_dad_know (6.14)*** **Not Equal 'Other [99]'**

6.15 other_know_biodad

Please specify:

Expects a single line text response (**required**)

6.16 bio_dad_rel

Is your biological father (birth father) married? (legally or traditionally married?)

Expects a single option response (**required**)

- Yes [1]
- No [0]
- I dont know [99]
- Other (Specify) [98]

Prerequisites
Skip when ***bio_dad_rel (6.16)*** **Not Equal 'Other (Specify) [98]'**

6.17 Other9_2

If Other , Please specify...

Expects a single line text response (**required**)

6.18 rest_hh

Who else lives in this household?

Expects multiple selected options (**required**)

- Sister [1]
- Brother [2]
- Uncle [3]
- Aunt [4]
- Grandmother [5]
- Grandfather [6]
- Stepfather [7]
- Stepmother [8]
- My own children [9]
- Guardian who is not related to you [10]
- Foster parent [11]
- Caregiver who is not related to you [12]
- No-one other than myself and my Caregiver [13]
- Spouse/Partner [14]

6.19 HH_SE_P

Household Socio-Economic Profile

6.20 water

I am now going to ask you a few questions related to items in this household and health of the household members.... Where is this household currently getting its water for drinking from?

Expects multiple selected options (**required**)

- Piped – inside house [1]
- Piped – yard [2]
- Piped – public [3]
- Borehole / well [4]
- River / stream [5]
- Water tanker (truck) [6]
- Rainwater tank [7]
- other (Specify) [98]

Prerequisites
Skip when ***water (6.20)*** **Excludes 'other (Specify) [98]'**

6.21 other4_2

If Other , please specify...

Expects a single line text response (**required**)

6.22 toilet

What type of toilet does the household use?

Expects a single option response (**required**)

- Flush toilet (own) [1]
- Flush toilet (shared with other household) [2]
- Bucket latrine [3]
- Pit latrine [4]
- Ventilated pit latrine [5]
- None [6]
- Other (Specify) [98]

Prerequisites
Skip when *toilet (6.22)* **Not Equal 'Other (Specify) [98]'**

6.23 other5_2

If Other, please specify

Expects a single line text response (**required**)

6.24 cook

What is the main source of fuel that the household uses to cook with?

Expects a single option response (**required**)

- Electricity [1]
- Gas [2]
- Paraffin [3]
- Charcoal/coal [4]
- Wood [5]
- Dung [6]
- Other (Specify) [98]

Prerequisites
Skip when *cook (6.24)* **Not Equal 'Other (Specify) [98]'**

6.25 Other6_2

If Other , please specify...

Expects a single line text response (**required**)

6.26 appli

Does the household have any of these items in working condition? Select all that apply...

Expects multiple selected options (**required**)

- Refrigerator [1]
- Radio [2]
- Television [3]
- Stove [4]
- Cell phone [5]
- Land line telephone [6]
- Car [7]
- Computer [8]
- Internet [9]
- Washing machine [10]
- Electricity [11]

6.27 income

Where does the income come from to take care of the household needs (food, water and electricity bills)? (You may choose more than one option)

Expects multiple selected options (**required**)

- My income [1]
- The household head's income [2]
- My mother's income [3]
- My father's income [4]
- Child support grant [5]
- Foster care grant [6]
- Care dependency grant [7]
- Disability grant [8]
- Pension [9]
- Family members send money [10]
- Other (Specify) [98]

Prerequisites
Skip when ***income (6.27)*** **Excludes 'Other (Specify) [98]'**

6.28 Other7_2

If Other , please specify...

Expects a single line text response (**required**)

6.29 no_food

In the past four weeks, how often was there no food to eat of any kind in your house because of lack of money?

Expects a single option response (**required**)

- Once [1]
- Twice [2]
- Three times [3]
- More than three times [4]
- Never [6]

6.30 no_food_sleep

In the past four weeks, how often did you or any other member of your household go to sleep hungry because of lack of food?

Expects a single option response (**required**)

- Once [1]
- Twice [2]
- Three times [3]
- More than three times [4]
- Never [5]

6.31 no_food_day

In the past four weeks, how often did you or any other member of your household go a whole day and night without eating because of lack of food?

Expects a single option response (**required**)

- Once [1]
- Twice [2]
- Three times [3]
- More than three times [4]
- Never [5]

6.32 tb_2

In the past year, how many individuals in this this household have had TB?

Expects a single option response (**required**)

- Some HouseHold members [1]
- No HouseHold members [2]
- Prefer Not to Say [97]
- Don't Know [99]

Branches

If response **Equals 'Don't Know [99]'** then skip to ***Resilience (7.1)***

If response **Equals 'Prefer Not to Say [97]'** then skip to ***Resilience (7.1)***

If response **Equals 'No HouseHold members [2]'** then skip to ***Resilience (7.1)***

6.33 tb_under_5

How many individuals in this this household that were diagnosed with TB were under the age of 5 years old?

Expects a numeric response (**required**)

6.34 tb_5_11

How many individuals in this this household that were diagnosed with TB were between 5-11 years old?

Expects a numeric response (**required**)

6.35 tb_12_19

How many individuals in this this household that were diagnosed with TB were between 12-19 years old?

Expects a numeric response (**required**)

6.36 tb_20

How many individuals in this this household that were diagnosed with TB were older than 20 years old?

Expects a numeric response (**required**)

Section 7. Resilience

7.1 Resilience

Listed below are a number of questions about you. These questions are designed to better understand how you cope with daily life and how you deal with daily challenges. Please indicate how much you agree with the following statements as they apply to you over the last MONTH. If a particular situation has not occurred recently, answer according to how you think you would have felt. (Please show the participant the scale card when answering the statements) Select NEXT to continue

---

7.2 adapt

I am able to adapt when changes occur.

Expects a single option response (**required**)

- Not true at all [1]
- Rarely true [2]
- Sometimes true [3]
- Often true [4]
- True nearly all the time [5]

7.3 deal

I can deal with whatever comes my way.

Expects a single option response (**required**)

- Not true at all [1]
- Rarely true [2]
- Sometimes true [3]
- Often true [4]
- True nearly all the time [5]

7.4 funny side

I try to see the funny side of things when I am faced with problems.

Expects a single option response (**required**)

- Not true at all [1]
- Rarely true [2]
- Sometimes true [3]
- Often true [4]
- True nearly all the time [5]

7.5 cope

Having to cope with stress can make me stronger.

Expects a single option response (**required**)

- Not true at all [1]
- Rarely true [2]
- Sometimes true [3]
- Often true [4]
- True nearly all the time [5]

7.6 bounce

I tend to bounce back after illness, injury, or other hardships.

Expects a single option response (**required**)

- Not true at all [1]
- Rarely true [2]
- Sometimes true [3]
- Often true [4]
- True nearly all the time [5]

7.7 goals

I believe I can achieve my goals, even if there are obstacles.

Expects a single option response (**required**)

- Not true at all [1]
- Rarely true [2]
- Sometimes true [3]
- Often true [4]
- True nearly all the time [5]

7.8 pressure

Under pressure, I stay focused and think clearly.

Expects a single option response (**required**)

- Not true at all [1]
- Rarely true [2]
- Sometimes true [3]
- Often true [4]
- True nearly all the time [5]

7.9 discouraged

I am not easily discouraged by failure.

Expects a single option response (**required**)

- Not true at all [1]
- Rarely true [2]
- Sometimes true [3]
- Often true [4]
- True nearly all the time [5]

7.10 strong

I think of myself as a strong person when dealing with life's challenges and difficulties.

Expects a single option response (**required**)

- Not true at all [1]
- Rarely true [2]
- Sometimes true [3]
- Often true [4]
- True nearly all the time [5]

7.11 handle

I am able to handle unpleasant or painful feelings like sadness, fear, and anger.

Expects a single option response (**required**)

- Not true at all [1]
- Rarely true [2]
- Sometimes true [3]
- Often true [4]
- True nearly all the time [5]

Section 8. Well-Being

8.1 wellbeing_intro

Next we'd like to get to know you a little better. Below are statements that you may agree or disagree. Using the scale below, tell me how much you agree or disagree with each statement. (Note to Fieldworker: Please show the participant the scale card for each question)

8.2 wellbeing_purpose

I lead a purposeful and meaningful life.

Expects a single option response (**required**)

- Strongly agree [1]
- Agree [2]
- Slightly agree [3]
- Neither agree nor disagree [4]
- Slightly disagree [5]
- Disagree [6]
- Strongly disagree [7]

8.3 wellbeing_social

My social relationships are supportive and rewarding.

Expects a single option response (**required**)

- Strongly agree [1]
- Agree [2]
- Slightly agree [3]
- Neither agree nor disagree [4]
- Slightly disagree [5]
- Disagree [6]
- Strongly disagree [7]

8.4 wellbeing_engaged

I am engaged and interested in my daily activities.

Expects a single option response (**required**)

- Strongly agree [1]
- Agree [2]
- Slightly agree [3]
- Neither agree nor disagree [4]
- Slightly disagree [5]
- Disagree [6]
- Strongly disagree [7]

8.5 wellbeing_contribute

I actively contribute to the happiness and well-being of others.

Expects a single option response (**required**)

- Strongly agree [1]
- Agree [2]
- Slightly agree [3]
- Neither agree nor disagree [4]
- Slightly disagree [5]
- Disagree [6]
- Strongly disagree [7]

8.6 wellbeing_competent

I am competent and capable in the activities that are important to me.

Expects a single option response (**required**)

- Strongly agree [1]
- Agree [2]
- Slightly agree [3]
- Neither agree nor disagree [4]
- Slightly disagree [5]
- Disagree [6]
- Strongly disagree [7]

8.7 wellbeing_good

I am a good person and live a good life.

Expects a single option response (**required**)

- Strongly agree [1]
- Agree [2]
- Slightly agree [3]
- Neither agree nor disagree [4]
- Slightly disagree [5]
- Disagree [6]
- Strongly disagree [7]

8.8 wellbeing_future

I am optimistic about my future.

Expects a single option response (**required**)

- Strongly agree [1]
- Agree [2]
- Slightly agree [3]
- Neither agree nor disagree [4]
- Slightly disagree [5]
- Disagree [6]
- Strongly disagree [7]

8.9 wellbeing_respect

People respect me.

Expects a single option response (**required**)

- Strongly agree [1]
- Agree [2]
- Slightly agree [3]
- Neither agree nor disagree [4]
- Slightly disagree [5]
- Disagree [6]
- Strongly disagree [7]

Section 9. General health, health, social services, and recreation

9.1 intro gen health

I am now going to ask you a few questions on health care or other care you may have received, and on your participation in clubs. Select NEXT to Continue.

9.2 TB

In the past year, have you been diagnosed with TB?

Expects a single option response (**required**)

- Yes [1]
- No [0]
- I prefer not to say [97]

9.3 health problem

In the past year, have you been to a clinic or hospital due to any other health problem?

Expects a single option response (**required**)

- Yes [1]
- No [0]

9.4 hospital

In the past year, did you ever stay in hospital?

Expects a single option response (**required**)

- Yes [1]
- No [0]

Branches

If response **Equals 'No [0]'** then skip to ***condom (9.8)***

9.5 hospitalstay

In the past year, when you stayed in hospital, how long did you stay in hospital?

Expects a single option response (**required**)

- Less than 1 week [1]
- 2-3 weeks [2]
- 1 month or more [3]

9.6 reasonhospitalstay

In the past year, when you stayed in hospital, what was the reason for your stay? (You may choose more than one option)

Expects multiple selected options (**required**)

- HIV-related [1]
- TB-related [2]
- Pregnancy-related [3]
- Asthma-related [4]
- Mental health-related [5]
- Diabetes [6]
- Injury [7]
- Other (Specify) [98]

Prerequisites
Skip when ***reasonhospitalstay (9.6)*** **Excludes 'Other (Specify) [98]'**

9.7 other5

if Other , Please specify...

Expects a single line text response (**required**)

9.8 condom

In the past year, did you get a condom from anyone or the following places: (You may select more than one option)

Expects multiple selected options (**required**)

- Clinic or hospital [1]
- School/University/college [2]
- Pharmacy [3]
- Friends [4]
- Parents [5]
- Family members [6]
- Boyfriend or girlfriend [7]
- Public toilets [8]
- Rise Club [9]
- Community health worker [10]
- War Rooms [11]
- I did not get a condom in the past year [12]
- Mobile van or truck [13]
- I prefer not to say [97]
- Other (Please specify) [98]

Prerequisites
Skip when ***condom (9.8)*** **Excludes 'Other (Please specify) [98]'**

9.9 other6

If Other , please specify ...

Expects a single line text response (**required**)

9.10 femalecondom

In the past year, did you get a FEMALE condom from anyone or the following places: (You may select more than one option)

Expects multiple selected options (**required**)

- Clinic or hospital [1]
- School/University/college [2]
- Pharmacy [3]
- Friends [4]
- Parents [5]
- Family members [6]
- Boyfriend or girlfriend [7]
- Public toilets [8]
- Mobile van or truck [9]
- I did not get a female condom within the past year [10]
- I prefer not to say [97]
- Other (Please specify) [98]

Prerequisites
Skip when ***femalecondom (9.10)*** **Excludes 'Other (Please specify) [98]'**

9.11 other7

if Other , Please specify...

Expects a single line text response (**required**)

9.12 contraception

In the past year, did you get any form of contraception/family planning (injection, implant, morning after pill, IUD, etc) from any of these places? (You may select more than one option)

Expects multiple selected options (**required**)

- Nurse or health worker at school [1]
- Nurse or health worker in a clinic or hospital [2]
- Mobile van/truck [3]
- Pharmacy [4]
- Nurse or health worker at home [5]
- I did not get contraception in the past year. [6]
- I prefer not to say [97]
- Other (Specify) [98]

Prerequisites
Skip when ***contraception (9.12)*** **Excludes 'Other (Specify) [98]'**

9.13 other8

if Other , Please specify...

Expects a single line text response (**required**)

9.14 prep

PrEP is a new prevention method in which people who do not have HIV take a pill daily to reduce their risk of becoming infected with HIV. Before today, have you ever heard of people who do not have HIV taking PrEP to keep from getting HIV?

Expects a single option response (**required**)

- Yes [1]
- No [0]
- I prefer not to say [97]

9.15 preptaken

In the past 12 months have you taken PrEP to keep you from getting HIV?

Expects a single option response (**required**)

- Yes [1]
- No [0]
- I prefer not to say [97]

9.16 counselor

In the past year, have you spoken to a counselor or social worker about the following? (You many choose more than one option)

Expects multiple selected options (**required**)

- Problems you experience [1]
- Getting an ID [2]
- Grants (child support grant, disability grant) [3]
- Abuse (abuse from a partner or family member or teacher or other adult) [4]
- Rape [5]
- None of the above [6]
- I prefer not to say [97]
- Other (Specify) [98]

Prerequisites
Skip when ***counselor (9.16)*** **Excludes 'Other (Specify) [98]'**

9.17 other_counselor

If other , Please specify...

Expects a single line text response (**required**)

9.18 help

Have you ever spoken to or received help from the police on the following?

Expects multiple selected options (**required**)

- Injury [1]
- Rape [2]
- None of the above [3]
- Other (Specify) [98]

Prerequisites
Skip when ***help (9.18)*** **Excludes 'Other (Specify) [98]'**

9.19 other_police

If Other , please specify...

Expects a single line text response (**required**)

9.20 pep

Post-exposure prophylaxis (or PEP) is a way to prevent HIV infection after sex or rape if you think the person might have given you HIV. It involves taking HIV medications as soon as possible (within 3 days) after sex or rape. Before today, had you ever heard of PEP?

Expects a single option response (**required**)

- Yes [1]
- No [0]

9.21 peptaken

Have you ever taken PEP?

Expects a single option response (**required**)

- Yes [1]
- No [0]
- I prefer not to say [97]

9.22 soulbuddys

Have you heard about Soul Buddyz Clubs? [Show picture of Logo] Soul Buddyz Clubs are clubs for children between 8 and 14 years, who spend time together to make a difference in their own lives and communities. They are usually based in schools.

Expects a single option response (**required**)

- Yes [1]
- No [0]

9.23 soul_magazine

Have you ever seen a Soul Buddyz magazine [Show picture of Soul Buddyz magazine]

Expects a single option response (**required**)

- Yes [1]
- No [0]

9.24 participated

Have you ever participated in a Soul Buddyz Club?

Expects a single option response (**required**)

- Yes [1]
- No [0]

9.25 member

Were you ever a member of a Soul Buddyz Club?

Expects a single option response (**required**)

- Yes [1]
- No [0]

9.26 freqattendance

How often have you attended a Soul Buddyz Club session?

Expects a single option response (**required**)

- 0 times [1]
- 1 time [2]
- 2 times [3]
- 3 times [4]
- 4 times [5]
- 5 times [6]
- 6 times [7]
- More than 6 times [8]

9.27 rise

Have you ever heard about Rise Clubs? [Show picture of Logo] Rise young womens clubs are clubs to empower young women to shape their own lives and the communities they live in.

Expects a single option response (**required**)

- Yes [1]
- No [0]

9.28 rise participation

Have you ever participated in a Rise Club?

Expects a single option response (**required**)

- Yes [1]
- No [0]

9.29 memberrise

Are you presently a member of a Rise Club?

Expects a single option response (**required**)

- Yes [1]
- No [0]

9.30 freqattendancerise

How often have you attended a Rise Club session?

Expects a single option response (**required**)

- 0 times [1]
- 1 time [2]
- 2 times [3]
- 3 times [4]
- 4 times [5]
- 5 times [6]
- 6 times [7]
- More than 6 times [8]

9.31 rise_mag

Have you ever seen a Rise Club Magazine? [Show picture of Rise Club Magazine]

Expects a single option response (**required**)

- Yes [1]
- No [0]

9.32 risetalk

Have you ever watched a television show called Rise Talk? [Show picture of Rise Talk hosts]

Expects a single option response (**required**)

- Yes [1]
- No [0]

9.33 flipchart

Have you ever attended a health education session at school where the facilitator used this flipchart? [Show picture of Keeping Girls in School flipchart]

Expects a single option response (**required**)

- Yes [1]
- No [0]

Prerequisites
Skip when ***province_Adolescent (1.3)*** **Not Equal 'Western Cape'**

9.34 wow

Have you heard about the Women of Worth program? [Show WoW logo]

Expects a single option response (**required**)

- Yes [1]
- No [0]

Prerequisites
Skip when ***province_Adolescent (1.3)*** **Not Equal 'Western Cape'**

9.35 part_wow

Have you ever participated in the Women of Worth program?

Expects a single option response (**required**)

- Yes [1]
- No [0]

Prerequisites
Skip when ***part_wow (9.35)*** **Equals 'No [0]'** OR
Skip when ***province_Adolescent (1.3)*** **Not Equal 'Western Cape'**

9.36 wow_incentive

Have you ever received a cash incentive because you participated in the Women of Worth program?

Expects a single option response (**required**)

- Yes [1]
- No [0]

Prerequisites
Skip when ***wow_incentive (9.36)*** **Equals 'No [0]'** OR
Skip when ***part_wow (9.35)*** **Equals 'No [0]'** OR
Skip when ***province_Adolescent (1.3)*** **Not Equal 'Western Cape'**

9.37 wow_cash

What did you do with the cash incentive?

Expects a single option response (**required**)

- Used it for food [1]
- Used it for clothing/entertainment/airtime [2]
- Saved it [3]
- Used it for my studies [4]
- It was stolen from me [5]
- I invested it in a business [6]
- Other (specify) [98]

Prerequisites
Skip when ***wow_cash (9.37)*** **Not Equal 'Other (specify) [98]'**

9.38 wow_other

If Other , Please specify...

Expects a single line text response (**required**)

9.39 hivinfo

Have you ever accessed information about sex or HIV online using a computer or phone?

Expects a single option response (**required**)

- Yes [1]
- No [0]
- I prefer not to say [97]

9.40 websites

What web sites have you accessed? (You may choose more than one option)

Expects multiple selected options (**required**)

- iLoveLife [1]
- Rise app [2]
- Soul Buddyz App [3]
- Chommy [4]
- BeWise [5]
- Momconnect [6]
- I have not accessed any of these websites [7]
- Other (Specify) [98]

Prerequisites
Skip when ***websites (9.40)*** **Excludes 'Other (Specify) [98]'**

9.41 Other9

If Other , please specify

Expects a single line text response (**required**)

9.42 SheConquers

Have you heard of a campaign called She Conquers?

Expects a single option response (**required**)

- Yes [1]
- No [0]

Section 10. Gender Equitable Men Scale (GEM)

10.1 gemintro

Now we will ask you a few questions about your opinions on men and women and relationships. We'd like you to tell us what you think. Remember there is no right or wrong answer. Select NEXT to continue

---

10.2 mandecides

It is the man who decides what type of sex to have.

Expects a single option response (**required**)

- Agree [1]
- Partially agree [2]
- Do not agree [3]

10.3 womanrole

A woman's most important role is to take care of her home and cook for her family.

Expects a single option response (**required**)

- Agree [1]
- Partially agree [2]
- Do not agree [3]

10.4 mansex

Men need sex more than women do.

Expects a single option response (**required**)

- Agree [1]
- Partially agree [2]
- Do not agree [3]

10.5 talksex

You dont talk about sex, you just do it.

Expects a single option response (**required**)

- Agree [1]
- Partially agree [2]
- Do not agree [3]

10.6 easy

Women who carry condoms on them are easy.

Expects a single option response (**required**)

- Agree [1]
- Partially agree [2]
- Do not agree [3]

10.7 otherwoman

A man needs other women, even if things with his wife are fine.

Expects a single option response (**required**)

- Agree [1]
- Partially agree [2]
- Do not agree [3]

10.8 bearen

There are times when a woman deserves to be beaten.

Expects a single option response (**required**)

- Agree [1]
- Partially agree [2]
- Do not agree [3]

10.9 resp

Changing diapers/nappies, giving the kids a bath, and feeding the kids are the mothers responsibility.

Expects a single option response (**required**)

- Agree [1]
- Partially agree [2]
- Do not agree [3]

10.10 avoid

It is a womans responsibility to avoid getting pregnant.

Expects a single option response (**required**)

- Agree [1]
- Partially agree [2]
- Do not agree [3]

10.11 finaldecision

A man should have the final word about decisions in his home.

Expects a single option response (**required**)

- Agree [1]
- Partially agree [2]
- Do not agree [3]

10.12 ready

Men are always ready to have sex.

Expects a single option response (**required**)

- Agree [1]
- Partially agree [2]
- Do not agree [3]

10.13 tolerate

A woman should tolerate violence in order to keep her family together.

Expects a single option response (**required**)

- Agree [1]
- Partially agree [2]
- Do not agree [3]

10.14 hit

If a woman cheats on a man, it is okay for him to hit her.

Expects a single option response (**required**)

- Agree [1]
- Partially agree [2]
- Do not agree [3]

10.15 hitnosex

It is okay for a man to hit his wife if she wont have sex with him.

Expects a single option response (**required**)

- Agree [1]
- Partially agree [2]
- Do not agree [3]

10.16 gay

I would never have a gay/lesbian friend.

Expects a single option response (**required**)

- Agree [1]
- Partially agree [2]
- Do not agree [3]

10.17 couple

A couple should decide together if they want to have children.

Expects a single option response (**required**)

- Agree [1]
- Partially agree [2]
- Do not agree [3]

10.18 suggest

In my opinion, a woman can suggest using condoms just like a man can.

Expects a single option response (**required**)

- Agree [1]
- Partially agree [2]
- Do not agree [3]

10.19 pregnantresp

If a guy gets a woman pregnant, the child is the responsibility of both.

Expects a single option response (**required**)

- Agree [1]
- Partially agree [2]
- Do not agree [3]

10.20 partner

A man should know what his partner likes during sex.

Expects a single option response (**required**)

- Agree [1]
- Partially agree [2]
- Do not agree [3]

10.21 father

It is important that a father is present in the lives of his children, even if he is no longer with the mother.

Expects a single option response (**required**)

- Agree [1]
- Partially agree [2]
- Do not agree [3]

10.22 contramanwoman

A man and a woman should decide together what type of contraceptive to use.

Expects a single option response (**required**)

- Agree [1]
- Partially agree [2]
- Do not agree [3]

10.23 friend

It is important to have a male friend that you can talk about your problems with.

Expects a single option response (**required**)

- Agree [1]
- Partially agree [2]
- Do not agree [3]

Section 11. Sexual Experiences

11.1 sexintro

NOTE TO FIELDWORKER For this section, the participant must answer each question privately without you seeing her response. We would now like to ask you some questions about your relationships and sexual history.We are going to focus on your relationships with boys and men. Some women have sexual relationships with women. However, almost all the question we will ask you now are about you relationships with boys and men. We will provide very detailed descriptions for some questions. We ask these questions so that we can learn as much about the experiences of young people like you today. I will read the questions to you and hand you the device so you can enter your answer privately. I will not know what your answer is. This will allow you to answer the questions in complete privacy. No one will be able to see the answers you put into the survey device. We appreciate you answering honestly and sharing as much information with us as possible. Select NEXT to continue

---

11.2 bf

In the past 12 months have you had a boyfriend or partner, even if only for a short time?

Expects a single option response (**required**)

- Yes [1]
- No [0]
- I prefer not to say [97]

11.3 date

In the past 12 months have you been on a date with a boy or man, even if only once?

Expects a single option response (**required**)

- Yes [1]
- No [0]
- I prefer not to say [97]

11.4 heavypetting

In the past 12 months have you engaged in heavy petting? By heavy petting we mean touching and fondling another persons private parts, or someone touching and fondling your private parts

Expects a single option response (**required**)

- Yes [1]
- No [0]
- I prefer not to say [97]

11.5 bf_circumcision

Thinking about your most recent boyfriend/partner, is he circumcised?

Expects a single option response (**required**)

- Yes [1]
- No [2]
- I don't know [3]
- I've never had a boyfriend [4]

11.6 age sex

Now Ill ask you questions on the FIRST TIME you had sex. (When we say had sex, we mean when the penis enters the vagina or anus/bum.) Sex can be something you agreed to, something you did not want to do, or were forced to do by someone you know such as a friend or family member or someone you dont know. The first time you had sex - how old were you?

Expects a single option response (**required**)

- Younger than 10 years old [1]
- 10 years old [2]
- 11 years old [3]
- 12 years old [4]
- 13 years old [5]
- 14 years old [6]
- 15 years old [7]
- 16 years old [8]
- 17 years old [9]
- 18 years old [10]
- 19 years old [11]
- Older than 19 [12]
- Never had sex (Virgin) [13]
- Prefer not to say [97]

Branches

If response **Not Equal 'Never had sex (Virgin) [13]'** then skip to ***first sexual partner (11.8)***

11.7 sex_conf

Sex is when a man/boy's penis enters a woman/girl's vagina or anus/bum. Sex can be something you agreed to, or something you did not want to do. No one, including family, friends, or neighbours, will know your answer to this question. Your answer will be kept a secret. We would like to ask you again just to be sure: have you ever had sex?

Expects a single option response (**required**)

- Yes, I have had sex [1]
- No, I have never had sex before [0]

Branches

If response **Equals 'No, I have never had sex before [0]'** then skip to ***transactional_sex (11.55)***

If response **Equals 'Yes, I have had sex [1]'** then skip to ***age sex (11.6)***

11.8 first sexual partner

The first time you had sex - who was it with?

Expects a single option response (**required**)

- Boyfriend (own age) [1]
- Boyfriend (+-5 years older) [2]
- Boyfriend (+-10 years older) [3]
- Blesser [4]
- Casual partner [5]
- Stranger [6]
- Father [7]
- Husband [8]
- Teacher [9]
- Other (Specify) [98]
- I prefer not to say [97]

Prerequisites
Skip when ***first sexual partner (11.8)*** **Not Equal 'Other (Specify) [98]'**

11.9 Other

If Other , Please specify...

Expects a single line text response (**required**)

11.10 partnerage

The first time you had sex - how old was the person you had sex with? If you are not sure or cant remember the of the age of the person, please guess their age. [NOTE TO FIELDWORKER] If a participant cant remember , doesnt know or prefers not to say , please enter 97 into the text box.

Expects a numeric response (**required**)

11.11 firstexp

The first time you had sex - was it (You may choose more than one option)

Expects multiple selected options (**required**)

- Something I wanted [1]
- Something I did not want [2]
- I was forced to against my will [3]
- I was raped [4]
- I prefer not to say [97]

11.12 firstfeel

Thinking about the first time you had sex - how do you feel about it now? (You may choose more than one option)

Expects multiple selected options (**required**)

- I wish I had waited longer before having sex [1]
- I wish I had not waited so long [2]
- It was the right time for me [3]
- It should not have happened at all [4]
- I prefer not to say [97]

11.13 firts condom

The first time you had sex - did you or the person you had sex with use a condom?

Expects a single option response (**required**)

- Yes [1]
- No [0]
- I prefer not to say [97]

11.14 preventpreg

The first time you had sex - did you use anything to prevent becoming pregnant? (You may choose more than one option)

Expects multiple selected options (**required**)

- I was on the injection [1]
- I was on the implant [2]
- I started contraception after my first sex act [3]
- I was on the pill [4]
- I took the morning after pill (emergency contraception) [5]
- I had a intra-uterine device (IUD) [6]
- I used a female condom [7]
- I had a diaphragm [8]
- I chose a time of my menstrual/ovulation cycle when I was unlikely to fall pregnant [9]
- I had had an operation to make me sterile [10]
- The person I had sex with used a male condom [11]
- The person I had sex with withdrew his penis during sex [12]
- The person I had sex with inserted his penis into my anus (bum) only [13]
- The person I was with had had an operation to make him sterile [14]
- Traditional medicine [15]
- I prefer not to say [97]
- Other (Specify) [98]
- I did not use anything [16]

Prerequisites
Skip when ***preventpreg (11.14)*** **Excludes 'Other (Specify) [98]'**

11.15 Other10

If Other , please specify

Expects a single line text response (**required**)

11.16 sex_more_once

Have you had sex more than once?

Expects a single option response (**required**)

- Yes [2]
- No [1]
- I prefer not to say [97]

Branches

If response **Equals 'No [1]'** then skip to ***male_partner intro (11.25)***

If response **Equals 'I prefer not to say [97]'** then skip to ***male_partner intro (11.25)***

11.17 lastage

Now think back to the LAST TIME you had sex (when the penis enters the vagina or anus/bum) [NOTE TO FIELDWORKER] If a participant cant remember , doesnt know or prefers not to say , please enter 97 into the text box. The last time you had sex - how old were you?

Expects a numeric response (**required**)

11.18 last sexual partner

The last time you had sex - who was it with?

Expects a single option response (**required**)

- Boyfriend (own age) [1]
- Boyfriend (+-5 years older) [2]
- Boyfriend (+-10 years older) [3]
- Blesser [4]
- Casual partner [5]
- Stranger [6]
- Father [7]
- Husband [8]
- Teacher [9]
- I prefer not to say [97]
- Other (Specify) [98]

Prerequisites
Skip when ***last sexual partner (11.18)*** **Not Equal 'Other (Specify) [98]'**

11.19 Other11

If Other , please specify

Expects a single line text response (**required**)

11.20 lastpartnerage

The last time you had sex - how old was the person you had sex with? If you are not sure of the age of the person, please guess their age. [NOTE TO FIELDWORKER] If a participant cant remember , doesnt know or prefers not to say , please enter 97 into the text box.

Expects a numeric response (**required**)

11.21 lastexp

The last time you had sex - (You may choose more than one option)

Expects multiple selected options (**required**)

- Something I wanted [1]
- Something I did not want [2]
- I was forced against my will [3]
- I was raped [4]
- I prefer not to say [97]

11.22 last_time

Thinking about the last time you had sex - how do you feel about it now?

Expects a single option response (**required**)

- It was the right time for me [1]
- It should not have happened at all [2]
- I prefer not to say [97]

11.23 last_time_condom

The last time you had sex - did you or the person you had sex with use a condom?

Expects a single option response (**required**)

- Yes [1]
- No [0]
- I prefer not to say [97]

11.24 last_prevent_preg

The last time you had sex - did you use anything to prevent becoming pregnant? (You may select more than one option) (You may choose more than one option)

Expects multiple selected options (**required**)

- I was on the injection [1]
- I was on the implant [2]
- I started contraception after my first sex act [3]
- I was on the pill [4]
- I took the morning after pill (emergency contraception) [5]
- I had a intra-uterine device (IUD) [6]
- I used a female condom [7]
- I had a diaphragm [8]
- I chose a time of my menstrual/ovulation cycle when I was unlikely to fall pregnant [9]
- I had had an operation to make me sterile [10]
- The person I had sex with used a male condom [11]
- The person I had sex with withdrew his penis during sex [12]
- The person I had sex with inserted his penis into my anus (bum) only [13]
- The person I was with had had an operation to make him sterile [14]
- Traditional medicine [15]
- I prefer not to say [97]
- I did not use anything [16]
- Other (Specify) [98]

11.25 male_partner intro

Now think about the past three months. I am now going to ask you about your male partners and about condom use in the past three months. We know it is sometimes difficult to use a condom every time you have sex Select Next to continue

---

11.26 number_girl_partners

In the past three months - how many girls or women have you had sex with? [NOTE TO FIELDWORKER] If a participant cant remember , doesnt know or prefers not to say , please enter 97 into the text box.

Expects a numeric response (**required**)

11.27 number_boy_partners

In the past three months - how many boys or men have you had sex with? [NOTE TO FIELDWORKER] If a participant cant remember , doesnt know or prefers not to say , please enter 97 into the text box.

Expects a numeric response (**required**)

Prerequisites
Skip when ***number_boy_partners (11.27)*** **Equals '0'** OR
Skip when ***number_boy_partners (11.27)*** **Equals '97'**

11.28 number_condom_use

The next questions are about when you had sex with boys or men: In the past three months - how many times have you had sex with a condom? [NOTE TO FIELDWORKER] If a participant cant remember , doesnt know or prefers not to say , please enter 97 into the text box.

Expects a numeric response (**required**)

11.29 number_without_condom

In the past three months - how many times have you had sex without a condom? [NOTE TO FIELDWORKER] If a participant cant remember , doesnt know or prefers not to say , please enter 97 into the text box.

Expects a numeric response (**required**)

11.30 number_casual

In the past three months - how many times have you had vaginal sex with CASUAL partner(s)? (Casual partners are people who are not your main partner) [NOTE TO FIELDWORKER] If a participant cant remember , doesnt know or prefers not to say , please enter 97 into the text box.

Expects a numeric response (**required**)

11.31 comdom_vaginal

In the past three months - how many times have you used a condom during vaginal sex with your casual partner(s)? [NOTE TO FIELDWORKER] If a participant cant remember , doesnt know or prefers not to say , please enter 97 into the text box.

Expects a numeric response (**required**)

11.32 vaginal_main

In the past three months - how many times have you had vaginal sex with MAIN partner(s)? [NOTE TO FIELDWORKER] If a participant cant remember , doesnt know or prefers not to say , please enter 97 into the text box.

Expects a numeric response (**required**)

Prerequisites
Skip when ***vaginal_main (11.32)*** **Equals '0'** OR
Skip when ***vaginal_main (11.32)*** **Equals '97'**

11.33 condom_vaginal_main

In the past three months - how many times have you used a condom during vaginal sex with your main partner(s)? [NOTE TO FIELDWORKER] If a participant cant remember , doesnt know or prefers not to say , please enter 97 into the text box.

Expects a numeric response (**required**)

11.34 number_vaginal_partners

In the past three months - how many VAGINAL sexual partners have you had? [NOTE TO FIELDWORKER] If a participant cant remember , doesnt know or prefers not to say , please enter 97 into the text box.

Expects a numeric response (**required**)

Branches

If response **Less Than '1'** then skip to ***use_condom_lst_3_monts (11.38)***

11.35 hiv_status_partner

Of these Q(**number_vaginal_partners (11.34)**) vaginal sex partners, how many were of HIV positive status? [NOTE TO FIELDWORKER] If a participant cant remember , doesnt know or prefers not to say , please enter 97 into the text box.

Expects a numeric response (**required**)

11.36 hiv_neg_partner

Of these Q(**number_vaginal_partners (11.34)**) vaginal sex partners, how many were of HIV negative status? [NOTE TO FIELDWORKER] If a participant cant remember , doesnt know or prefers not to say , please enter 97 into the text box.

Expects a numeric response (**required**)

11.37 status_unknown

Of these Q(**number_vaginal_partners (11.34)**) vaginal sex partners, how many were of unknown HIV status? [NOTE TO FIELDWORKER] If a participant cant remember , doesnt know or prefers not to say , please enter 97 into the text box.

Expects a numeric response (**required**)

11.38 use_condom_lst_3_monts

In the past three months have you used a condom?

Expects a single option response (**required**)

- Yes [1]
- No [0]

11.39 freq_condom_use

In the past three months how often did you use a condom with your casual partner(s)?

Expects a single option response (**required**)

- Always [1]
- Most of the time [2]
- Sometimes [3]
- Never [4]
- Prefer not to say [97]

11.40 freq_condom_main

In the past three months how often did you use a condom with your main partner(s)?

Expects a single option response (**required**)

- Always [1]
- Most of the time [2]
- Sometimes [3]
- Never [4]
- Prefer not to say [97]

11.41 casual_anal

In the past three months- How many times have you had anal sex with casual partner(s)? [NOTE TO FIELDWORKER] If a participant cant remember , doesnt know or prefers not to say , please enter 97 into the text box.

Expects a numeric response (**required**)

Prerequisites
Skip when ***casual_anal (11.41)*** **Less Than '1'** OR
Skip when ***casual_anal (11.41)*** **Equals '97'**

11.42 condom_anal_casual

In the past three months- How many times have you used a condom during anal sex with your casual partner(s)? [NOTE TO FIELDWORKER] If a participant cant remember , doesnt know or prefers not to say , please enter 97 into the text box.

Expects a numeric response (**required**)

11.43 anal_main

In the past three months- How many times have you had anal sex with your main partner(s)? [NOTE TO FIELDWORKER] If a participant cant remember , doesnt know or prefers not to say , please enter 97 into the text box.

Expects a numeric response (**required**)

Prerequisites
Skip when ***anal_main (11.43)*** **Less Than '1'** OR
Skip when ***anal_main (11.43)*** **Equals '97'**

11.44 Condom_anal_male

In the past three months- How many times have you used a condom during anal sex with your main partner(s)? [NOTE TO FIELDWORKER] If a participant cant remember , doesnt know or prefers not to say , please enter 97 into the text box.

Expects a numeric response (**required**)

11.45 number_anal

In the past three months- How many anal sexual partners have you had? [NOTE TO FIELDWORKER] If a participant cant remember , doesnt know or prefers not to say , please enter 97 into the text box.

Expects a numeric response (**required**)

Branches

If response **Less Than '1'** then skip to ***boys_total (11.49)***

If response **Equals '97'** then skip to ***boys_total (11.49)***

11.46 hiv_pos_anal

If you had anal intercourse, how many of your anal sex partners were of HIV positive status? [NOTE TO FIELDWORKER] If a participant cant remember , doesnt know or prefers not to say , please enter 97 into the text box.

Expects a numeric response (**required**)

11.47 hiv_neg_anal

If you had anal intercourse, how many of your anal sex partners were of HIV negative status? [NOTE TO FIELDWORKER] If a participant cant remember , doesnt know or prefers not to say , please enter 97 into the text box.

Expects a numeric response (**required**)

11.48 anal_unknown_status

If you had anal intercourse, how many of your anal sex partners were of HIV unknown status? [NOTE TO FIELDWORKER] If a participant cant remember , doesnt know or prefers not to say , please enter 97 into the text box.

Expects a numeric response (**required**)

11.49 boys_total

I am now going to ask you about your sexual experiences in the past year: In the past year- how many boys or men have you had sex with? [NOTE TO FIELDWORKER] If a participant cant remember , doesnt know or prefers not to say , please enter 97 into the text box.

Expects a numeric response (**required**)

Branches

If response **Less Than '1'** then skip to ***partner_older_5 (11.53)***

11.50 total_boys_main

Think of all the boys or men you had sex with in the past year: how many were main partners? [NOTE TO FIELDWORKER] If a participant cant remember , doesnt know or prefers not to say , please enter 97 into the text box.

Expects a numeric response (**required**)

11.51 total_boys_casual

Think of all the boys or men you had sex with in the past year: how many were casual partners? [NOTE TO FIELDWORKER] If a participant cant remember , doesnt know or prefers not to say , please enter 97 into the text box.

Expects a numeric response (**required**)

11.52 total_onceoff

Think of all the boys or men you had sex with in the past year: how many were once-off partners (you had sex with them only once)? [NOTE TO FIELDWORKER] If a participant cant remember , doesnt know or prefers not to say , please enter 97 into the text box.

Expects a numeric response (**required**)

11.53 partner_older_5

In the past year have any of your sexual partners been five or more years older than you?

Expects a single option response (**required**)

- Yes [1]
- No [0]

11.54 Partners_hiv_pos

In the past year have any of your sexual partners been HIV positive?

Expects a single option response (**required**)

- Yes [1]
- No [0]
- I dont know [99]
- Prefer not to say [97]

11.55 transactional_sex

Have you ever GIVEN oral, anal, or vaginal sex to someone because you expected to get or got any of these things? (You may choose more than one option)

Expects multiple selected options (**required**)

- Money [1]
- Transport [2]
- Food for myself and/or my family [3]
- Clothes or shoes [4]
- Shelter [5]
- School fees/school uniforms [6]
- Airtime [7]
- Cellphone [8]
- Items for children or family [9]
- Cosmetics [10]
- I have not done this [11]
- I prefer not to say [97]
- Other (Specify) [98]

Prerequisites
Skip when ***transactional_sex (11.55)*** **Excludes 'Other (Specify) [98]'**

11.56 other_trans_sex

If Other , please specify ...

Expects a single line text response (**required**)

11.57 transactional_relationship

In the past 12 months have you started or stayed in a relationship with a man or boy so that you could receive any of the following? (You may choose more than one option) (You may choose more than one option)

Expects multiple selected options (**required**)

- Money [1]
- Transport [2]
- Food for myself and/or my family [3]
- Clothes or shoes [4]
- Shelter [5]
- School fees/school uniforms [6]
- Airtime [7]
- Cellphone [8]
- Items for children or family [9]
- Cosmetics [10]
- I have not done this [11]
- I prefer not to say [97]
- Other (Specify) [98]

Prerequisites
Skip when ***transactional_relationship (11.57)*** **Excludes 'Other (Specify) [98]'**

11.58 other_trans_rel

If Other , please specify....

Expects a single line text response (**required**)

Section Prerequisites
Skip when *sex_conf (11.7)* Equals'No, I have never had sex before [0]'

Section 12. Pregnancy and abortion history

12.1 peg_intro

NOTE TO FIELDWORKER For this section, the participant must answer each question privately without you seeing her response

---

12.2 preg

Now we would like to find out about any pregnancies you might have had. Have you ever been pregnant?

Expects a single option response (**required**)

- Yes [1]
- No [0]
- I prefer not to say [97]

Branches

If response **Equals 'No [0]'** then skip to ***contraceptives (12.15)***

If response **Equals 'I prefer not to say [97]'** then skip to ***contraceptives (12.15)***

12.3 age_preg

How old were you when you first became pregnant? [NOTE TO FIELDWORKER] If a participant cant remember , doesnt know or prefers not to say , please enter 97 into the text box.

Expects a numeric response (**required**)

12.4 preg_want

When you first became pregnant, did you want a baby?

Expects a single option response (**required**)

- Yes [1]
- No [0]
- I prefer not to say [97]

12.5 numerous_preg

Have you been pregnant more than once?

Expects a single option response (**required**)

- Yes [1]
- No [0]
- I prefer not to say [97]

12.6 times_preg

How many times have you been pregnant? [NOTE TO FIELDWORKER] If a participant cant remember , doesnt know or prefers not to say , please enter 97 into the text box.

Expects a numeric response (**required**)

12.7 living_children

How many living children do you have? [NOTE TO FIELDWORKER] If a participant cant remember , doesnt know or prefers not to say , please enter 97 into the text box.

Expects a numeric response (**required**)

Prerequisites
Skip when ***times_preg (12.6)*** **Less Than '2'**

12.8 last_preg_want

The last time you were pregnant, did you want a baby?

Expects a single option response (**required**)

- Yes [1]
- No [0]
- I prefer not to say [97]

12.9 last_preg

What happened with your last pregnancy?

Expects a single option response (**required**)

- I gave birth to a healthy baby [1]
- I had a miscarriage [2]
- I had an abortion [3]
- I gave birth to a baby but it was not alive when it came out [4]
- The baby was born and then died a few days or weeks or months later [5]
- I took herbs or muti to end my pregnancy [6]
- I prefer not to say [97]
- Other (Specify) [98]

Prerequisites
Skip when ***last_preg (12.9)*** **Not Equal 'Other (Specify) [98]'**

12.10 Other12

If Other , please specify

Expects a single line text response (**required**)

12.11 preg_school

The last time you were pregnant, did you stay in school during your pregnancy?

Expects a single option response (**required**)

- Yes [1]
- No [2]
- I had already left school [3]
- I prefer not to say [97]

12.12 abortion

Did you ever choose to have an abortion?

Expects a single option response (**required**)

- Yes [1]
- No [0]
- I prefer not to say [97]

Branches

If response **Equals 'No [0]'** then skip to ***contraceptives (12.15)***

If response **Equals 'I prefer not to say [97]'** then skip to ***contraceptives (12.15)***

12.13 abortion_facility

The last time you had an abortion where did you get it?

Expects a single option response (**required**)

- Clinic or hospital [1]
- Marie Stopes clinic [2]
- Traditional doctor or sangoma or inyanga [3]
- Doctor that advertised on Google or on a flyer in the community [4]
- Private doctor [5]
- I prefer not to say [97]
- Other (Specify) [98]

Prerequisites
Skip when ***abortion_facility (12.13)*** **Not Equal 'Other (Specify) [98]'**

12.14 Other13

If Other , please specify

Expects a single line text response (**required**)

12.15 contraceptives

Some girls or young womn take steps to prevent having a baby during vaginal sex (this is when a guy inserts his penis into a girls vagina). Lets talk more about this. Have you ever taken or used contraception or something else to prevent you from getting pregnant?

Expects a single option response (**required**)

- Yes [1]
- No [0]
- I prefer not to say [97]

12.16 prevent_now

If you have taken or done anything to prevent pregnancy . . . What are you taking now to prevent pregnancy? (You may choose more than one option) (You may choose more than one option)

Expects multiple selected options (**required**)

- I am on the injection [1]
- I am on the implant [2]
- I started contraception after my first sex act [3]
- I am on the pill [4]
- I take the morning after pill (emergency contraception) [5]
- I have a intra-uterine device (IUD) [6]
- I use a female condom [7]
- I have a diaphragm [8]
- I choose a time of my menstrual/ovulation cycle when I am unlikely to fall pregnant [9]
- I have had an operation to make me sterile [10]
- The person I have sex with uses a male condom [11]
- The person I have sex with withdraws his penis during sex before ejaculating [12]
- The person I have sex with inserts his penis into my anus (bum) only [13]
- The person I am with has had an operation to make him sterile [14]
- I use traditional medicine [15]
- I prefer not to say [97]
- I am not using anything now [16]
- Other (Specify) [98]

Prerequisites
Skip when *prevent_now (12.16)* Excludes 'Other (Specify) [98]'

12.17 Other14

If Other , please specify

Expects a single line text response (**required**)

12.18 preg_partner

Are you or your partner currently trying to get pregnant?

Expects a single option response (**required**)

- Yes [1]
- No [0]
- I prefer not to say [97]

12.19 emergency_contraceptive

Have you ever heard of emergency contraception? Emergency contraception refers to pills that may be taken after sex, to prevent pregnancy.

Expects a single option response (**required**)

- Yes [1]
- No [0]
- I prefer not to say [97]

12.20 taken_emerg_contraceptive

Have you ever taken emergency contraception?

Expects a single option response (**required**)

- Yes [1]
- No [0]
- I prefer not to say [97]

12.21 methods

Have you heard of any of the following ways that a couple can protect themselves if one partner is HIV+ and the other partner is HIV-, but they want to have a child? If yes, you may choose more than one option. If no, select I have never heard of these methods.

Expects multiple selected options (**required**)

- The HIV+ partner can go on antiretroviral medications (ARVs) for HIV [1]
- The HIV- partner can go on ARVs to protect themselves from HIV infection (also known as pre-exposure prophylaxis or PrEP) [2]
- The couple can time sex to when a woman is most likely to conceive [3]
- The couple can have sex with a condom EXCEPT during time periods when a woman is most likely to conceive [4]
- The mother can lower the risk of transmission to the child (for example by electing to deliver via C-section or other 'safe delivery' options) [5]
- If pregnant and HIV+, the mother can lower the risk of transmission by taking ARVs during her pregnancy [6]
- I have never heard of these methods [7]

12.22 problems

What are the problems you face in getting any FAMILY PLANNING method (implant, pill, morning after pill, condom, IUD) to prevent pregnancy? (You may choose more than one option)

Expects multiple selected options (**required**)

- I don't have money for transport [1]
- I have to travel a long distance to the clinic [2]
- The clinic is open only during school hours [3]
- The clinic has long waiting queues [4]
- I cannot take time off from work or family responsibilities to go to the clinic [5]
- I do not like the way staff treat me at the clinic [6]
- I am scared someone will notice me at the clinic getting these items [7]
- My family prevents me from getting a method [8]
- I don't know where to get a method [9]
- I do not have any problems [10]
- My partner prevents me from getting a method [11]
- I prefer not to say [97]
- Other (Specify) [98]

Prerequisites
Skip when ***problems (12.22)*** **Excludes 'Other (Specify) [98]'**

12.23 Other15

If Other , please specify

Expects a single line text response (**required**)

12.24 easier

What makes it easier for you to get any family planning method (implant, pill, morning after pill, condom, iud) to prevent pregnancy? (you may choose more than one option)

Expects multiple selected options (**required**)

- I can get transport to the clinic [1]
- The clinic is close by [2]
- My school gives me time off to attend the clinic [3]
- The clinic has short queues [4]
- The staff at the clinic treat me well [5]
- My school gives me these items [6]
- My friends/family give me these items [7]
- I prefer not to say [97]
- Nothing makes it easier for me [8]
- Other (Specify) [98]

Prerequisites
Skip when ***easier (12.24)*** **Excludes 'Other (Specify) [98]'**

12.25 Other16

If Other , please specify

Expects a single line text response (**required**)

Section 13. HIV testing history, ART history

13.1 hiv_instruct

NOTE TO FIELDWORKER For this next section, the participant must answer each question privately without you seeing her response.

13.2 hiv_test

Do you know what HIV is? Well, it is a virus that infects the immune system in the body and many people and families in South Africa are living with HIV. I am now going to ask you some questions about this. Lets go... Have you ever had an HIV test?

Expects a single option response (**required**)

- Yes [1]
- No [0]

Branches

If response **Equals 'No [0]'** then skip to ***reason_no_test (13.26)***

13.3 freq_hiv_test

Approximately how often do you have an HIV test?

Expects a single option response (**required**)

- Every three to five months [1]
- Every six months [2]
- Every year [3]
- Every two years [4]
- Less often [5]
- I prefer not to say [97]

13.4 mobile_test_service

Have you ever had an HIV test in a mobile health service?

Expects a single option response (**required**)

- Yes [1]
- No [0]

13.5 last_year

Did you have an HIV test in the past year?

Expects a single option response (**required**)

- Yes [1]
- No [0]
- I prefer not to say [97]

13.6 where_test

Where did you have your last HIV test done?

Expects a single option response (**required**)

- Clinic or hospital [1]
- Youth centre [2]
- School [3]
- At work [4]
- In a mobile van [5]
- In my community [6]
- At home [7]
- Private doctor [8]
- Traditional healer [9]
- Tent [10]
- Other (specify) [98]

Prerequisites
Skip when *where_test (13.6)* **Not Equal 'Other (specify) [98]'**

13.7 other_hiv

If Other , Please specify

Expects a single line text response (**required**)

13.8 result

Did you ever get your HIV result?

Expects a single option response (**required**)

- Yes [1]
- No [0]

Branches

If response **Equals 'No [0]'** then skip to ***chances (13.10)***

13.9 hiv_result

The last time you tested, what was your HIV test result?

Expects a single option response (**required**)

- HIV- positive [1]
- HIV- negative [2]
- I did not get my result [3]
- Prefer not to say [97]

Prerequisites
Skip when ***hiv_result (13.9)*** **Equals 'HIV- positive [1]'**

13.10 chances

Thinking about your current behaviors, how high are the chances you could get HIV?

Expects a single option response (**required**)

- Low: 0-10% [1]
- Moderate: 11-30% [2]
- High: 31-60% [3]
- Very high: above 60% [4]
- Unsure [5]

Prerequisites
Skip when ***hiv_result (13.9)*** **Equals 'HIV- positive [1]'**

13.11 confidence

How confident are you that you can stay HIV-negative in your lifetime?

Expects a single option response (**required**)

- Highly confident (above 60% sure I'll stay HIV-negative) [1]
- Confident (31- 60% sure I'll stay HIV-negative) [2]
- Moderately confident (11-30% I will stay HIV-negative) [3]
- Low confidence (0-10% sure I will stay HIV-negative) [4]
- Unsure [5]

Prerequisites
Skip when ***hiv_result (13.9)*** **Equals 'HIV- negative [2]'** OR
Skip when ***hiv_result (13.9)*** **Equals 'I did not get my result [3]'** OR
Skip when ***hiv_result (13.9)*** **Equals 'Prefer not to say [97]'** OR
Skip when ***result (13.8)*** **Equals 'No [0]'**

13.12 told_people

Have you told any of these people that you are HIV-positive? (You may choose more than one option)

Expects multiple selected options (**required**)

- ☐ Boyfriend/partner [1]
- ☐ Parent/caregiver [2]
- ☐ Other family members [3]
- ☐ Friends [4]
- ☐ Teacher /lecturer [5]
- ☐ Sports coach [6]
- ☐ Principal [7]
- ☐ School nurse [8]
- ☐ Nurse at a clinic or hospital [9]
- ☐ Counsellor or social worker [10]
- ☐ I did not tell anyone [11]
- ☐ I prefer not to say [97]
- ☐ Other (Specify) [98]

Prerequisites
Skip when ***told_people (13.12)*** **Excludes 'Other (Specify) [98]'**

13.13 Other17

If Other , please specify

Expects a single line text response (**required**)

Prerequisites
Skip when ***hiv_result (13.9)*** **Equals 'HIV- negative [2]'** OR
Skip when ***hiv_result (13.9)*** **Equals 'I did not get my result [3]'** OR
Skip when ***hiv_result (13.9)*** **Equals 'Prefer not to say [97]'** OR
Skip when ***result (13.8)*** **Equals 'No [0]'**

13.14 support

Which of these people give you support for your HIV? (You may choose more than one option)

Expects multiple selected options (**required**)

- ☐ Boyfriend/partner [1]
- ☐ Parent/caregiver [2]
- ☐ Other family members [3]
- ☐ Friends [4]
- ☐ Teacher /lecturer [5]
- ☐ Sports coach [6]
- ☐ Principal [7]
- ☐ School nurse [8]
- ☐ Nurse at a clinic or hospital [9]
- ☐ Counsellor or social worker [10]
- ☐ I did not tell anyone [11]
- ☐ No one gives me support [12]
- ☐ Other (Specify) [98]

Prerequisites
Skip when ***support (13.14)*** **Excludes 'Other (Specify) [98]'**

13.15 Other18

If Other , please specify

Expects a single line text response (**required**)

Prerequisites
Skip when ***hiv_result (13.9)*** **Equals 'HIV- negative [2]'** OR
Skip when ***hiv_result (13.9)*** **Equals 'I did not get my result [3]'** OR
Skip when ***hiv_result (13.9)*** **Equals 'Prefer not to say [97]'** OR
Skip when ***result (13.8)*** **Equals 'No [0]'**

13.16 bad_treatment

Which of these people treat you badly because you are HIV-positive? (You may choose more than one option)

Expects multiple selected options (**required**)

- Boyfriend/partner [1]
- Parent/caregiver [2]
- Other family members [3]
- Friends [4]
- Teacher /lecturer [5]
- Sports coach [6]
- Principal [7]
- School nurse [8]
- Nurse at a clinic or hospital [9]
- Counsellor or social worker [10]
- I did not tell anyone [11]
- No one treats me badly [12]
- I prefer not to say [97]
- Other (Specify) [98]

Prerequisites
Skip when ***bad_treatment (13.16)*** **Excludes 'Other (Specify) [98]'**

13.17 Other19

If Other , please specify

Expects a single line text response (**required**)

Prerequisites
Skip when ***hiv_result (13.9)*** **Equals 'HIV- negative [2]'** OR
Skip when ***hiv_result (13.9)*** **Equals 'I did not get my result [3]'** OR
Skip when ***hiv_result (13.9)*** **Equals 'Prefer not to say [97]'** OR
Skip when ***result (13.8)*** **Equals 'No [0]'**

13.18 arvs

Have you ever taken antiretrovirals (ARVs)?

Expects a single option response (**required**)

- Yes [1]
- No [0]
- I prefer not to say [97]

Branches

If response **Equals 'No [0]'** then skip to ***not_taking_arv (13.24)***

Prerequisites
Skip when ***hiv_result (13.9)*** **Equals 'HIV- negative [2]'** OR
Skip when ***hiv_result (13.9)*** **Equals 'I did not get my result [3]'** OR
Skip when ***hiv_result (13.9)*** **Equals 'Prefer not to say [97]'** OR
Skip when ***result (13.8)*** **Equals 'No [0]'**

13.19 taking_arvs

Are you still taking ARVs now?

Expects a single option response (**required**)

- Yes [1]
- No [0]
- Prefer not to say [97]

Prerequisites
Skip when ***hiv_result (13.9)*** **Equals 'HIV- negative [2]'** OR
Skip when ***hiv_result (13.9)*** **Equals 'I did not get my result [3]'** OR
Skip when ***hiv_result (13.9)*** **Equals 'Prefer not to say [97]'** OR
Skip when ***result (13.8)*** **Equals 'No [0]'**

13.20 miss_app

In the past year, did you miss any of your clinic appointments for your HIV care?

Expects a single option response (**required**)

- Yes [1]
- No [0]

Prerequisites
Skip when ***hiv_result (13.9)*** **Equals 'HIV- negative [2]'** OR
Skip when ***hiv_result (13.9)*** **Equals 'I did not get my result [3]'** OR
Skip when ***hiv_result (13.9)*** **Equals 'Prefer not to say [97]'** OR
Skip when ***result (13.8)*** **Equals 'No [0]'**

13.21 problem_meds

What are the problems you faced in going to the clinic for your for HIV care or appointments for HIV care? (You may choose more than one option)

Expects multiple selected options (**required**)

- I have transport problems [1]
- I have to travel a long distance to the clinic [2]
- The clinic is open only during school/work hours [3]
- The clinic has long waiting queues [4]
- I do not like the way staff treat me at the clinic [5]
- I am scared someone will notice me at the clinic [6]
- I do not have anyone to take me to the clinic [7]
- I have no problems [8]
- Other (specify) [98]
- I prefer not to say [97]

Prerequisites
Skip when ***problem_meds (13.21)*** **Excludes 'Other (specify) [98]'**

13.22 Other21

If Other , please specify

Expects a single line text response (**required**)

Prerequisites
Skip when ***hiv_result (13.9)*** **Equals 'HIV- negative [2]'** OR
Skip when ***hiv_result (13.9)*** **Equals 'I did not get my result [3]'** OR
Skip when ***hiv_result (13.9)*** **Equals 'Prefer not to say [97]'** OR
Skip when ***result (13.8)*** **Equals 'No [0]'**

13.23 easier_clinic_app

What helps you in going to the clinic for your appointments for your HIV care? (You may choose more than one option)

Expects multiple selected options (**required**)

- ☐ I can get transport to the clinic [1]
- ☐ The clinic is close by [2]
- ☐ My school/work gives me time off to attend my appointments [3]
- ☐ The clinic has short queues [4]
- ☐ The staff at the clinic treat me well [5]
- ☐ My parent or guardian or caregiver comes with me to the clinic [6]
- ☐ I have friends or family member who support me in going to the clinic [7]
- ☐ I dont get any help [8]
- ☐ Other (Specify) [98]

Prerequisites
Skip when ***hiv_result (13.9)*** **Equals 'HIV- negative [2]'** OR
Skip when ***hiv_result (13.9)*** **Equals 'I did not get my result [3]'** OR
Skip when ***hiv_result (13.9)*** **Equals 'Prefer not to say [97]'** OR
Skip when ***arvs (13.18)*** **Equals 'Yes [1]'** OR
Skip when ***result (13.8)*** **Equals 'No [0]'**

13.24 not_taking_arv

Why are you not taking ARVs? (You may choose more than one option)

Expects multiple selected options (**required**)

- ☐ My CD4 count is high [1]
- ☐ I'm scared the people I live with will find out [2]
- ☐ I am using traditional medication or herbs or muti or medication from the sangoma or inyanga [3]
- ☐ I do not like taking them [4]
- ☐ I do not need them [5]
- ☐ I feel healthy [6]
- ☐ Someone told me to stop taking them [7]
- ☐ My supply ran out [8]
- ☐ I prefer not to say [97]
- ☐ Other (Specify) [98]

Prerequisites
Skip when ***not_taking_arv (13.24)*** **Excludes 'Other (Specify) [98]'**

13.25 Other20

If Other , please specify

Expects a single line text response (**required**)

Prerequisites
Skip when *hiv_test (13.2)* Equals 'Yes [1]'

13.26 reason_no_test

What are your reasons for not having ever had an HIV test? (You may choose more than one option)

Expects multiple selected options (**required**)

- I do not know where to get tested [1]
- I do not think that I have HIV [2]
- I am not at risk for HIV [3]
- I trust my partner [4]
- I was afraid to find out that I might be HIV positive [5]
- I am not ready to have an HIV test [6]
- I was concerned about confidentiality [7]
- I was concerned about stigma, discrimination or rejection [8]
- I was concerned about losing my job [9]
- I am concerned about the standard of service [10]
- I haven’t got around to it [11]
- I prefer not to say [97]
- Other (Specify) [98]

Prerequisites
Skip when *reason_no_test (13.26)* Excludes 'Other (Specify) [98]'

13.27 Other23

If Other, please specify.

Expects a single line text response (**required**)

Section 14. Sexually transmitted infections history

14.1 sti

NOTE TO FIELDWORKER For this next section, the participant must answer each question privately without you seeing her response. People sometimes get infections around their private areas (penis, vagina, anus (bum), we are calling these sexually transmitted infections (STIs)) Lets talk more about this... Did a doctor or nurse ever say you had a sexually transmitted infection (STI) in or around your private parts (penis, vagina, anus/butt)?

Expects a single option response (**required**)

- Yes [1]
- No [0]
- I prefer not to say [97]

14.2 sti_exp

In the past year, did you experience any of the following on your private areas (penis, vagina, anus/butt)? (You may choose more than one option)

Expects multiple selected options (**required**)

- Itching [1]
- Lumps [2]
- Sores or warts or rash [3]
- Redness [4]
- Unusual smell from yellow or brown or white discharge [5]
- Pain or burning feeling when urinating or having sex [6]
- No- I do not have any of these issues listed above on my private areas [7]
- I prefer not to say [97]

14.3 sti_treatment

If you experienced any one of the following on your private areas in the past year, where did you go to get treatment? Itching or lumps or sores or warts or rash or redness or unusual smell from yellow or brown or white substance or pain or burning feeling when urinating or having sex)

Expects a single option response (**required**)

- Clinic or hospital [1]
- School nurse [2]
- Doctor [3]
- Traditional healer [4]
- Pharmacy [5]
- I did not get treatment [6]
- I did not experience symptoms [7]
- I prefer not to say [97]
- Other (Specify) [98]

Prerequisites
Skip when ***sti_treatment (14.3)*** **Not Equal 'Other (Specify) [98]'**

14.4 Other24

If Other, please specify.

Expects a single line text response (**required**)

14.5 problem_sti_treatment

What are the problems you face in getting treatment for STIs? (You may choose more than one option) (You may choose more than one option) (You may choose more than one option)

Expects multiple selected options (**required**)

- [ ] I have transport problems [1]
- [ ] I have to travel a long distance to the clinic [2]
- [ ] The clinic is open only during school/work hours [3]
- [ ] The clinic has long waiting queues [4]
- [ ] I do not like the way staff treat me at the clinic [5]
- [ ] I am scared someone will notice me at the clinic [6]
- [ ] I do not have anyone to take me to the clinic [7]
- [ ] I have no problems [8]
- [ ] I have not sought treatment for STIs [9]
- [ ] Other (specify) [98]

Prerequisites
Skip when ***problem_sti_treatment (14.5)*** **Excludes 'Other (specify) [98]'**

14.6 Other25

If Other , please specify

Expects a single line text response (**required**)

14.7 sti_help

What helps you get treatment for STIs? (You may choose more than one option) (You may choose more than one option)

Expects multiple selected options (**required**)

- [ ] I can get transport to the clinic [1]
- [ ] The clinic is close by [2]
- [ ] My school/work gives me time off to attend my appointments [3]
- [ ] The clinic has short queues [4]
- [ ] The staff at the clinic treat me well [5]
- [ ] My parent or guardian or caregiver comes with me to the clinic [6]
- [ ] I have friends or family member who support me in going to the clinic [7]
- [ ] I have not sought treatment for STIs [8]
- [ ] Other (Specify) [98]

Prerequisites
Skip when ***sti_help (14.7)*** **Excludes 'Other (Specify) [98]'**

14.8 Other26

If Other , please specify

Expects a single line text response (**required**)

Section 15. Sexual and reproductive health knowledge and beliefs

15.1 instruc_sex_health

NOTE TO FIELDWORKER For this next section, the participant must answer each question privately without you seeing her response. We are now going to talk about sexual health, and ask you on your opinion on a few statements. These are just AGREE or DISAGREE questions. You need to tell me whether you AGREE or DISAGREE with the statements. Dont worry it is not a test Lets go!....

15.2 rep_health_intro

Emergency contraception or morning after pill , is a pill a girl can get from a clinic to prevent pregnancy if she and her partner did not use protection (condom or other method)

Expects a single option response (**required**)

- Agree [1]
- Disagree [2]

15.3 witch_craft

A person can get HIV or AIDS because of witchcraft.

Expects a single option response (**required**)

- Agree [1]
- Disagree [2]

15.4 virgin

A guy who has HIV or AIDS can be cured by having sex with a girl who is a virgin

Expects a single option response (**required**)

- Agree [1]
- Disagree [2]

15.5 circum

If a guy is circumcised there is no chance he could get HIV

Expects a single option response (**required**)

- Agree [1]
- Disagree [2]

15.6 mother_to_child

Antiretrovirals (ARVs) given to a pregnant women can prevent her from passing HIV to her baby

Expects a single option response (**required**)

- Agree [1]
- Disagree [2]

15.7 anal

If you have anal sex (when a penis enters the bum) with someone who is HIV-positive, you can become infected with HIV

Expects a single option response (**required**)

- Agree [1]
- Disagree [2]

Section 16. Intimate partner violence (IPV)

16.1 instruc_prt_violence

NOTE TO FIELDWORKER For this next section, the participant must answer each question privately without you seeing her response.

16.2 ipv_insult

Sometimes couples argue, these arguments may get out of control and get violent. The next few questions are about what you have experienced in your relationships. In the past year, has a boyfriend or partner insulted you or made you feel bad about yourself? How often has this happened?

Expects a single option response (**required**)

- Never [1]
- Once [2]
- More than Once [3]
- I havent had a boyfriend or partner in the last 12 months [4]
- I prefer not to say [97]

Branches

If response **Equals 'I havent had a boyfriend or partner in the last 12 months [4]'** then skip to ***ipv_forced (16.12)***

16.3 ipv_humiliated

In the past year, has a boyfriend or partner made fun of or humiliated you in front of other people? How often has this happened?

Expects a single option response (**required**)

- Never [1]
- Once [2]
- More than Once [3]
- I havent had a boyfriend or partner in the last 12 months [4]
- I prefer not to say [97]

16.4 ipv_scare

In the past year, has a boyfriend or partner threatened to hurt you or done things to scare or intimidate you on purpose. For example by the way he looked at you, by yelling and smashing things? How often has it happened?

Expects a single option response (**required**)

- Never [1]
- Once [2]
- More than Once [3]
- I havent had a boyfriend or partner in the last 12 months [4]
- I prefer not to say [97]

16.5 ipv_slapped

In the past year, has a boyfriend or partner slapped you or thrown something at you which could hurt you? How often has this happened?

Expects a single option response (**required**)

- Never [1]
- Once [2]
- More than Once [3]
- I havent had a boyfriend or partner in the last 12 months [4]
- I prefer not to say [97]

16.6 ipv_pushed

In the past year, has a boyfriend or partner pushed or shoved you? How often has this happened?

Expects a single option response (**required**)

- Never [1]
- Once [2]
- More than Once [3]
- I havent had a boyfriend or partner in the last 12 months [4]
- I prefer not to say [97]

16.7 ipv_hit

In the past year, has a boyfriend or partner hit you with a fist or with something else which could hurt you? How often has this happened?

Expects a single option response (**required**)

- Never [1]
- Once [2]
- More than Once [3]
- I havent had a boyfriend or partner in the last 12 months [4]
- I prefer not to say [97]

16.8 ipv_kicked

In the past year, has a boyfriend or partner kicked, dragged, beat, choked or burned you? How often has this happened?

Expects a single option response (**required**)

- Never [1]
- Once [2]
- More than Once [3]
- I havent had a boyfriend or partner in the last 12 months [4]
- I prefer not to say [97]

16.9 ipv_gun

In the past year, has a boyfriend or partner threatened to use or actually used a gun, knife or other weapon against you? How often has this happened?

Expects a single option response (**required**)

- Never [1]
- Once [2]
- More than Once [3]
- I havent had a boyfriend or partner in the last 12 months [4]
- I prefer not to say [97]

16.10 ipv_threat

In the past year, have you had sex with a boyfriend or partner when you didn't want to because he forced or threatened or pressured you or because you were unable to stop him?

Expects a single option response (**required**)

- Never [1]
- Once [2]
- More than Once [3]
- I havent had a boyfriend or partner in the last 12 months [4]
- I prefer not to say [97]

16.11 ipv_sex

In the past year, have you had sex because your boyfriend or partner threatened to go out with or have sex with someone else if you didnt have sexual relations with him?

Expects a single option response (**required**)

- Never [1]
- Once [2]
- More than Once [3]
- I havent had a boyfriend or partner in the last 12 months [4]
- I prefer not to say [97]

16.12 ipv_forced

Now we will be asking you about boyfriends/partners AND ALSO people who were not your boyfriend or partner. Have you EVER had sex with a man because you were forced or had sex with him because you were threatened?

Expects a single option response (**required**)

- Never [1]
- Once [2]
- More than Once [3]
- I prefer not to say [97]

16.13 rape

Have you ever been raped?

Expects a single option response (**required**)

- Yes [1]
- No [2]
- Prefer not to answer [97]

Branches

If response **Equals 'No [2]'** then skip to ***mspss_intro (17.1)***

16.14 ipv_help

The next questions are about getting help when you experienced violence or rape by your boyfriend or someone who was not your boyfriend. Did you ever get any help with your experiences of violence or rape from a boyfriend or a boy or man who was not your boyfriend.

Expects a single option response (**required**)

- Yes [1]
- No [0]
- I have never experienced violence or rape from a boyfriend or other man [2]
- I prefer not to say [97]

Prerequisites
Skip when *ipv_help (16.14)* **Not Equal 'Yes [1]'**

16.15 ipv_help_services

When you experienced violence or rape from a boyfriend or someone who was not a boyfriend, did you ever get help from any of the following? (You may choose more than one option)

Expects multiple selected options (**required**)

- Police [1]
- Hospital or clinic [2]
- Social workers or counsellors [3]
- Lawyers or legal advice [4]
- Parent [5]
- Shelter for women and girls [6]
- Religious leaders [7]
- Other leaders in your community [8]
- Thuthuzela Care Centre [9]
- Organisation/NGO [10]
- Community health worker [11]
- Teacher [12]
- Friend [13]
- I prefer not to say [97]
- I did not seek help [14]
- Other (Specify) [98]

Prerequisites
Skip when *ipv_help_services (16.15)* **Excludes 'Other (Specify) [98]'**

16.16 Other27

If Other , please specify

Expects a single line text response (**required**)

Prerequisites
Skip when *ipv_help (16.14)* **Not Equal 'Yes [1]'**

16.17 ipv_help_services2

The last time you experienced violence or rape from a boyfriend or someone who was not a boyfriend, did you ever get help from any of the following? (You may choose more than one option)

Expects multiple selected options (**required**)

- Police [1]
- Hospital or clinic [2]
- Social workers or counsellors [3]
- Lawyers or legal advice [4]
- Parent [5]
- Shelter for women and girls [6]
- Religious leaders [7]
- Other leaders in your community [8]
- Thuthuzela Care Centre [9]
- Organisation/NGO [10]
- Community health worker [11]
- Teacher [12]
- Friend [13]
- I prefer not to say [97]
- Other (Specify) [98]
- I did not seek help [14]

Prerequisites
Skip when ***ipv_help_services2 (16.17)*** **Excludes 'Other (Specify) [98]'**

16.18 Other28

If Other , please specify

Expects a single line text response (**required**)

Section 17. Social Support (MSPSS)

17.1 mspss_intro

For this section, the field worker may resume entering the responses Now we want to learn who you turn to for support. We are interested in how you feel about the following statements. Indicate how you feel about each statement. Let's Go!

17.2 mspss_special_person

There is a special person around when I am in need...

Expects a single option response (**required**)

- Very strongly disagree [1]
- Strongly disagree [2]
- Mildly disagree [3]
- Neutral [4]
- Mildly agree [5]
- Strongly agree [6]
- Very strongly agree [7]

17.3 mspss_sorrow

There is a special person with whom I can share my joys and sorrows.

Expects a single option response (**required**)

- Very strongly disagree [1]
- Strongly disagree [2]
- Mildly disagree [3]
- Neutral [4]
- Mildly agree [5]
- Strongly agree [6]
- Very strongly agree [7]

17.4 mspss_help

My family really tries to help me

Expects a single option response (**required**)

- Very strongly disagree [1]
- Strongly disagree [2]
- Mildly disagree [3]
- Neutral [4]
- Mildly agree [5]
- Strongly agree [6]
- Very strongly agree [7]

17.5 mspss_family

I get the emotional help and support I need from my family.

Expects a single option response (**required**)

- Very strongly disagree [1]
- Strongly disagree [2]
- Mildly disagree [3]
- Neutral [4]
- Mildly agree [5]
- Strongly agree [6]
- Very strongly agree [7]

17.6 mspss_special

I have a special person who is a real source of comfort to me.

Expects a single option response (**required**)

- Very strongly disagree [1]
- Strongly disagree [2]
- Mildly disagree [3]
- Neutral [4]
- Mildly agree [5]
- Strongly agree [6]
- Very strongly agree [7]

17.7 mspss_friends

My friends really try to help me.

Expects a single option response (**required**)

- Very strongly disagree [1]
- Strongly disagree [2]
- Mildly disagree [3]
- Neutral [4]
- Mildly agree [5]
- Strongly agree [6]
- Very strongly agree [7]

17.8 mspss_count_on

I can count on my friends when things go wrong.

Expects a single option response (**required**)

- Very strongly disagree [1]
- Strongly disagree [2]
- Mildly disagree [3]
- Neutral [4]
- Mildly agree [5]
- Strongly agree [6]
- Very strongly agree [7]

17.9 mspss_talk

I can talk about my problems with my family.

Expects a single option response (**required**)

- Very strongly disagree [1]
- Strongly disagree [2]
- Mildly disagree [3]
- Neutral [4]
- Mildly agree [5]
- Strongly agree [6]
- Very strongly agree [7]

17.10 mspss_friend_sorrow

I have friends with whom I can share my joys and sorrows.

Expects a single option response (**required**)

- Very strongly disagree [1]
- Strongly disagree [2]
- Mildly disagree [3]
- Neutral [4]
- Mildly agree [5]
- Strongly agree [6]
- Very strongly agree [7]

17.11 mspss_feelings

There is a special person in my life who cares about my feelings.

Expects a single option response (**required**)

- Very strongly disagree [1]
- Strongly disagree [2]
- Mildly disagree [3]
- Neutral [4]
- Mildly agree [5]
- Strongly agree [6]
- Very strongly agree [7]

17.12 mspss_decisions

My family is willing to help me make decisions.

Expects a single option response (**required**)

- Very strongly disagree [1]
- Strongly disagree [2]
- Mildly disagree [3]
- Neutral [4]
- Mildly agree [5]
- Strongly agree [6]
- Very strongly agree [7]

17.13 mspss_problems

I can talk about my problems with my friends.

Expects a single option response (**required**)

- Very strongly disagree [1]
- Strongly disagree [2]
- Mildly disagree [3]
- Neutral [4]
- Mildly agree [5]
- Strongly agree [6]
- Very strongly agree [7]

Section 18. Social Assessment (SASCAT)

18.1 sascat_intro

We are now going to look at your involvement in community groups.

18.2 sascat_member

In the last 12 months, have you been a member of the following types of groups or clubs in your area?

Expects multiple selected options (**required**)

- Club [1]
- Sports group [2]
- Youth/student group [3]
- Community group [4]
- Religious group (church, Sunday school) [5]
- Volunteer group to assist the sick/elderly/poor [6]
- Political group [7]
- None of the above [8]
- Other (Specify) [98]

Prerequisites
Skip when ***sascat_member (18.2)*** **Excludes 'Other (Specify) [98]'**

18.3 Other29

If Other , please specify

Expects a single line text response (**required**)

Prerequisites
Skip when ***sascat_member (18.2)*** **Includes 'None of the above [8]'**

18.4 sascat_involv

In the last 12 months, how would you describe your involvement in the group or club?

Expects multiple selected options (**required**)

- Received a loan or their form of financial support [1]
- Attended meetings [2]
- Participated in discussions on various topics [3]
- Attended training [4]
- Participated in decision making [5]
- Served as a leader of a group [6]
- Other (please specify) [98]

Prerequisites
Skip when ***sascat_involv (18.4)*** **Excludes 'Other (please specify) [98]'**

18.5 other30

If Other, please specify...

Expects a single line text response (**required**)

18.6 sascat_help

In the last 12 months, did you receive from the group/club any emotional help, economic help or assistance in helping you know or do things? You may choose more than one option.

Expects multiple selected options (**required**)

- Club [1]
- Youth/student group [2]
- Community group [3]
- Religious group (church, Sunday school) [4]
- Volunteer group to assist the sick/elderly/poor [5]
- Political group [6]
- None of the above [7]
- Other (Specify) [98]

Prerequisites
Skip when ***sascat_help (18.6)*** **Excludes 'Other (Specify) [98]'**

18.7 other31

If other , please specify ...

Expects a single line text response (**required**)

18.8 sascat_support

In the last 12 months, have you received any help or support from any of the following, this can be emotional help, economic help or assistance in helping you know or do things? (You may choose more than one option)

Expects multiple selected options (**required**)

- Family [1]
- Neighbours [2]
- Friends who are not neighbours [3]
- Community leaders [4]
- Religious leaders [5]
- Politicians [6]
- Government officials/civil service [7]
- Charitable organisations/NGO [8]
- None of the Above [9]
- Other (Specify) [98]

Prerequisites
Skip when ***sascat_support (18.8)*** **Excludes 'Other (Specify) [98]'**

18.9 other32

If Other , please specify ...

Expects a single line text response (**required**)

18.10 sascat_community

In the last 12 months, have you joined together with other community members to address a problem or common issue?

Expects a single option response (**required**)

- Yes [1]
- No [0]

18.11 sascat_authority

In the last 12 months, have you talked with a local authority or governmental organisation about problems in this community?

Expects a single option response (**required**)

- Yes [1]
- No [0]

18.12 taking_trusted

In general, can the majority of people in this community be trusted?

Expects a single option response (**required**)

- Yes [1]
- No [0]

18.13 sascat_get_along

Do the majority of people in this community generally get along with each other?

Expects a single option response (**required**)

- Yes [1]
- No [0]

18.14 sascat_apart

Do you feel as though you are really a part of this community?

Expects a single option response (**required**)

- Yes [1]
- No [0]

18.15 sascat_advantage

Do you think that the majority of people in this community would try to take advantage of you if they got the chance?

Expects a single option response (**required**)

- Yes [1]
- No [0]

Section 19. Substance abuse

19.1 sub_intro

NOTE TO FIELDWORKER For this section, the participant must answer each question privately without you seeing her response Now we will ask you about drinking alcohol:

19.2 Substance_often

How often do you have a drink containing alcohol?

Expects a single option response (**required**)

- Never [1]
- Monthly or less [2]
- 2-4 times a month [3]
- 2-3 times a week [4]
- 4 or more times a week [5]
- I prefer not to say [97]

Branches

If response **Equals ' Never [1]'** then skip to ***drug_intro (19.6)***

19.3 std_drinks

How many standard drinks containing alcohol do you have on a typical day? A standard drink is a can of beer, or a small glass of wine, or a tot of whisky or brandy. A cider is two standard drinks.

Expects a single option response (**required**)

- 1 or 2 [1]
- 3 or 4 [2]
- 5 or 6 [3]
- 7 to 9 [4]
- 10 or more [5]
- I do not drink alcohol [6]
- I prefer not to say [97]

19.4 six_or_more

How often do you have six or more drinks on one occasion?

Expects a single option response (**required**)

- Never [1]
- Less than monthly [2]
- Monthly [3]
- Weekly [4]
- Daily or almost daily [5]
- I prefer not to say [97]

Prerequisites
Skip when ***sex_conf (11.7)*** **Equals 'No, I have never had sex before [0]'**

19.5 sex_drunk

Have you ever had sex while you were drunk?

Expects a single option response (**required**)

- Never [1]
- Once [2]
- More than once [3]
- I prefer not to say [97]

19.6 drug_intro

Now we want to ask about drugs. Drugs include: cannabis (marijuana, hash, hash oil); Nyope; Whoonga, amphetamines (Tik, methamphetamine, phenmetraline, khat, betel nut, ritaline); cocaine (crack, freebase, coca leaves); opiates (smoked heroin, heroin, opium); hallucinogens (ecstasy, LSD, mescaline, peyote, PCP/angel dust, psilocybin, DMT); solvents/inhalants (thinner, trichlorethylene, gasoline/petrol, gas, solution, glue); GHB and others (GHB, anabolic steroids, laughing gas, amyl nitrate, anticholinergic compounds). Medication counts as drugs when you take them more often than the doctor has prescribed; you take them to have fun, feel good, get 'high', or wonder what sort of effect they have on you; if you receive them from a relative or a friend; if you bought them on the 'black market' or they were stolen. Please know that you will not get in trouble , please answer these questions honestly. Your answers will be kept private.

19.7 alcohol_freq

How often do you use drugs other than alcohol?

Expects a single option response (**required**)

- Never [1]
- Once a month or less often [2]
- 2-4 times a month [3]
- 2-3 times a week [4]
- 4 or more times a week [5]
- I prefer not to say [97]

Branches

If response **Equals ' Never [1]'** then skip to ***whonga (19.18)***

19.8 drug_use

How often do you use more than one type of drug on the same occasion?

Expects a single option response (**required**)

- Never [1]
- Once a month or less often [2]
- 2-4 times a month [3]
- 2-3 times a week [4]
- 4 or more times a week [5]
- I prefer not to say [97]

19.9 influ

How often are you influenced heavily by drugs?

Expects a single option response (**required**)

- Never [1]
- Less often than once a month [2]
- Every month [3]
- Every week [4]
- Daily or almost every day [5]
- I prefer not to say [97]

Prerequisites
Skip when ***influ (19.9)*** **Equals 'Never [1]'** OR
Skip when ***influ (19.9)*** **Equals 'I prefer not to say [97]'**

19.10 freq_used_day

How many times do you take drugs on a typical day when you use drugs?

Expects a single option response (**required**)

19.10 freq_used_day

How many times do you take drugs on a typical day when you use drugs?

Expects a single option response (**required**)

- 0 times a day [1]
- 1-2 times a day [2]
- 3-4 times a day [3]
- 5-6 times a day [4]

- 7 or more times aday [5]
- I prefer not to say [97]

19.11 longing

Over the past year, how often have you have you felt that your longing for drugs was so strong that you could not resist it?

Expects a single option response (**required**)

- Never [1]
- Less often than once a month [2]
- Every month [3]
- Every week [4]
- Daily or almost every day [5]

19.12 not_stop

Over the past year, how often has it happened that you have not been able to stop taking drugs once you started?

Expects a single option response (**required**)

- Never [1]
- Less often than once a month [2]
- Every month [3]
- Every week [4]
- Daily or almost every day [5]

19.13 neglect

How often over the past year have you taken drugs and then neglected to do something you should have done?

Expects a single option response (**required**)

- Never [1]
- Less often than once a month [2]
- Every month [3]
- Every week [4]
- Daily or almost every day [5]

19.14 morning_after

How often over the past year have you needed to take a drug the morning after heavy drug use the day before?

Expects a single option response (**required**)

- Never [1]
- Less often than once a month [2]
- Every month [3]
- Every week [4]
- Daily or almost every day [5]

19.15 guilt

How often over the past year have you had guilt feelings or a bad conscience because you used drugs?

Expects a single option response (**required**)

- Never [1]
- Less often than once a month [2]
- Every month [3]
- Every week [4]
- Daily or almost every day [5]

19.16 hurt

Have you or anyone else been hurt (mentally or physically) because you used drugs?

Expects a single option response (**required**)

- Yes, but not over the past year [1]

- Yes, over the past year [2]
- No [0]

19.17 worried

Has a relative or friend, a doctor or a nurse, or anyone else, been worried about your drug use or said to you that you should stop using drugs?

Expects a single option response (**required**)

- Yes, but not over the past year [1]
- Yes, over the past year [2]
- No [0]

19.18 whonga

Have you or someone you know ever used ARVs to get high OR another mixture of substances that you suspect may have contained ARVs to get high (this mix is sometimes called nyaope or whoonga)? (You may choose more than one option)

Expects multiple selected options (**required**)

- I have done this [1]
- Someone I know has done this [2]
- Neither I nor someone I know has done this [3]

Prerequisites
Skip when ***whonga (19.18)*** **Includes 'Neither I nor someone I know has done this [3]'**

19.19 arv_high

How have YOU used ARVs or mixtures of substances that you suspect may have contained ARVS to get high? (You may choose more than one option)

Expects multiple selected options (**required**)

- Smoked [1]
- Snorted [2]
- Injected [3]
- Swallowed [4]
- Inserted/absorbed [5]
- I have not taken ARV's to get high [6]

Prerequisites
Skip when ***sex_conf (11.7)*** **Equals 'No, I have never had sex before [0]'**

19.20 sex_drugs

In the past 3 months, when you have had vaginal or anal sex, how often were YOU using alcohol or other drugs?

Expects a single option response (**required**)

- Never [1]
- Less than half the time [2]
- About half the time [3]
- More than half the time [4]
- Always [5]

Prerequisites
Skip when ***sex_conf (11.7)*** **Equals 'No, I have never had sex before [0]'**

19.21 sex_drugs_partner

In the past 3 months, when you have had vaginal or anal sex, how often was your PARTNER using alcohol or other drugs?

Expects a single option response (**required**)

- Never [1]
- Less than half the time [2]
- About half the time [3]

- More than half the time [4]
- Always [5]

19.22 sexual

In the past 3 months, has alcohol or drugs led you to do something sexual?

Expects a single option response (**required**)

- Yes [1]
- Yes, more than once [2]
- No [0]

Prerequisites
Skip when ***sex_conf (11.7)*** **Equals 'No, I have never had sex before [0]'**

19.23 sex_unplanned_drugs

In the past 3 months, have you had sex when you did not plan to, because you had been drinking or using drugs?

Expects a single option response (**required**)

- Yes [1]
- Yes, more than once [2]
- No [0]

Prerequisites
Skip when ***sex_conf (11.7)*** **Equals 'No, I have never had sex before [0]'**

19.24 without_condom

In the past 3 months, did you have sex without a condom because you were drinking or using drugs?

Expects a single option response (**required**)

- Yes [1]
- Yes, more than once [2]

- No [0]

Section 20. HIV Stigma

20.1 intro_stigma

In The next section we will ask you about your feelings towards people with HIV and AIDS. Please answer whether you agree or disagree with the following statements. **NOTE TO FIELDWORKER** For this section, the participant must answer each question privately, without you seeing her response.

20.2 hiv_status

Do you know your HIV status?

Expects a single option response (**required**)

- Yes, I am HIV positive [1]
- Yes, I am HIV negative [2]
- No, I do not know my status [3]
- Prefer not to say [97]

Branches

If response **Equals 'Yes, I am HIV positive [1]'** then skip to ***difficult (20.12)***

20.3 dirty_hiv

People who have HIV/AIDS are dirty.

Expects a single option response (**required**)

- Agree [1]
- Disagree [2]

20.4 cursed

People who have HIV/AIDS are cursed.

Expects a single option response (**required**)

- Agree [1]
- Disagree [2]

20.5 ashamed

People who have HIV/AIDS should be ashamed.

Expects a single option response (**required**)

- Agree [1]
- Disagree [2]

20.6 safe

It is safe for people who have HIV/AIDS to work with children.

Expects a single option response (**required**)

- Agree [1]
- Disagree [2]

20.7 restrictions

People with HIV/AIDS must expect some restrictions on their freedom.

Expects a single option response (**required**)

- Agree [1]
- Disagree [2]

20.8 wrong

A person with HIV/AIDS must have done something wrong and deserves to be punished.

Expects a single option response (**required**)

Agree [1]

Disagree [2]

20.9 isolated

People who have HIV/AIDS should be isolated.

Expects a single option response (**required**)

Agree [1]

Disagree [2]

20.10 friends

I do not want to be friends with someone who has HIV/AIDS.

Expects a single option response (**required**)

Agree [1]

Disagree [2]

20.11 work

People who have HIV/AIDS should not be allowed to work.

Expects a single option response (**required**)

Agree [1]

Disagree [2]

Prerequisites
Skip when ***hiv_status (20.2)*** **Equals 'Yes, I am HIV negative [2]'** OR
Skip when ***hiv_status (20.2)*** **Equals 'No, I do not know my status [3]'** OR
Skip when ***hiv_status (20.2)*** **Equals 'Prefer not to say [97]'**

20.12 difficult

Please tell us how you feel about living with HIV: do you agree or disagree? It is difficult to tell people about my HIV infection.

Expects a single option response (**required**)

Agree [1]

Disagree [2]

Prerequisites
Skip when ***hiv_status (20.2)*** **Equals 'Yes, I am HIV negative [2]'** OR
Skip when ***hiv_status (20.2)*** **Equals 'No, I do not know my status [3]'** OR
Skip when ***hiv_status (20.2)*** **Equals 'Prefer not to say [97]'**

20.13 dirty

Being HIV positive makes me feel dirty.

Expects a single option response (**required**)

Agree [1]

Disagree [2]

Prerequisites
Skip when ***hiv_status (20.2)*** **Equals 'Yes, I am HIV negative [2]'** OR
Skip when ***hiv_status (20.2)*** **Equals 'No, I do not know my status [3]'** OR
Skip when ***hiv_status (20.2)*** **Equals 'Prefer not to say [97]'**

20.14 feel_guilty

I feel guilty because I am HIV positive.

Expects a single option response (**required**)

Agree [1]

Disagree [2]

Prerequisites
Skip when ***hiv_status (20.2)*** **Equals 'Yes, I am HIV negative [2]'** OR
Skip when ***hiv_status (20.2)*** **Equals 'No, I do not know my status [3]'** OR
Skip when ***hiv_status (20.2)*** **Equals 'Prefer not to say [97]'**

20.15 feel_ashamed

I am ashamed because I am HIV positive.

Expects a single option response (**required**)

Agree [1]

Disagree [2]

Prerequisites
Skip when ***hiv_status (20.2)*** **Equals 'Yes, I am HIV negative [2]'** OR
Skip when ***hiv_status (20.2)*** **Equals 'No, I do not know my status [3]'** OR
Skip when ***hiv_status (20.2)*** **Equals 'Prefer not to say [97]'**

20.16 worthless

I sometimes feel worthless because I am HIV positive.

Expects a single option response (**required**)

Agree [1]

Disagree [2]

Prerequisites
Skip when ***hiv_status (20.2)*** **Equals 'Yes, I am HIV negative [2]'** OR
Skip when ***hiv_status (20.2)*** **Equals 'No, I do not know my status [3]'** OR
Skip when ***hiv_status (20.2)*** **Equals 'Prefer not to say [97]'**

20.17 hide

I hide my HIV status from others.

Expects a single option response (**required**)

Agree [1]

☐ Disagree [2]

Section 21. End

21.1 end_intro

Well done!!!! You have completed this questionnaire. I certainly appreciate you taking the time out today to complete this. I really enjoyed my time with you today The information you have given me is going to be helpful in improving HIV prevention and care programmes and health services for young people in your community and in South Africa Did you enjoy completing this questionnaire?

Expects a single option response (**required**)

☐ Yes [1]

☐ No [0]

Prerequisites
Skip when ***end_intro (21.1)*** **Equals 'No [0]'**

21.2 end_yes

If YES... (You may select more than one option)

Expects multiple selected options (**required**)

☐ I had fun completing it [1]

☐ I learnt a lot [2]

☐ I understood the question [3]

☐ I was comfortable sharing this info with the HERStory study [4]

☐ It felt good to be open or honest about myself [5]

☐ It helped me to share the bad things I've been through [6]

☐ It made me think about my life [7]

☐ This research will help others [8]

☐ It helped me realize I need help [9]

☐ It made me feel somebody cares about what I've been through [10]

☐ It made it easier for me to ask for help [11]

Prerequisites
Skip when ***end_intro (21.1)*** **Equals 'Yes [1]'**

21.3 end_no

If NO.... (You may select more than one option)

Expects multiple selected options (**required**)

☐ It was too long [1]

☐ I did not understand a lot of the questions [2]

☐ I felt uncomfortable sharing personal info with the HERStory study [3]

☐ It made me feel distressed (sad, upset, angry) [4]

☐ I didn't like thinking about the bad things I've been through [5]

21.4 share

Is there anything else you would like to share with us?

Expects a long text response (optional)

21.5 accept_home_test

After this questionnaire, you will be offered a rapid HIV test. If you accept, you will receive your results within 15 minutes of the test. Would you like to have the HIV test now?

Expects a single option response (**required**)

- Yes, I would like be tested to know if I am HIV positive or HIV negative [1]
- No, I would not like to be tested [2]

Prerequisites
Skip when ***accept_home_test (21.5)*** **Equals 'Yes, I would like be tested to know if I am HIV positive or HIV negative [1]'**

21.6 reason_home_test_refusal

What is the main reason why you prefer not to have an HIV test now?

Expects a single option response (**required**)

- I already know my status, HIV positive [1]
- I already know my status, HIV negative [2]
- I don't want to be tested at home [3]
- Other (please specify) [98]

Prerequisites
Skip when ***reason_home_test_refusal (21.6)*** **Not Equal 'Other (please specify) [98]'**

21.7 other_specify

If other , Please specify.

Expects a single line text response (**required**)

21.8 lab_test_result

We will send the blood we have taken from you to the laboratory for testing. In two weeks time, we will send your HIV test results to the nearest clinic for you to collect. How do you feel about going to the clinic to receive your laboratory HIV test results?

Expects a single option response (**required**)

- I wish to collect my results from the clinic [1]
- I am unsure if I will collect my results from the clinic [2]

- [ ] I do not wish to know my results and I will not go to the clinic [3]

Section 22. Referrals

22.1 intro_referral

LASTLY - This section is to be completed only to report a participant referral.

22.2 referral

Fieldworker: Is a participant Referral required?

Expects a single option response (**required**)

- [ ] Yes - referral required [1]
- [ ] No [0]

Branches

If response **Equals 'No [0]'** then skip to ***end (23.1)***

22.3 referral_type

Type of referral:

Expects a single option response (**required**)

- [ ] Clinical [1]
- [ ] Psychosocial [2]
- [ ] Other [98]

Prerequisites
Skip when ***referral_type (22.3)*** **Not Equal 'Other [98]'**

22.4 other_referral_type

Please specify type of referral:

Expects a single line text response (**required**)

22.5 referred_centre

Specific Referred Center:

Expects a single line text response (**required**)

22.6 referral_comments

Please provide any other comments with regards to the referral:

Expects a single line text response (**required**)

Section 23. Upload Submission

23.1 end

Remember, if you have any questions or worries please talk to the fieldworker on the study before you leave or contact us on the number on your information sheet Be strong, healthy and happy and have a good day!! Press next to upload submission.
