# Supplementary material for: Factors influencing adolescent girls and young women’s participation in a combination HIV prevention intervention in South Africa
Source: BMC Public Health. 2021 Feb 27;21:417. doi: 10.1186/s12889-021-10462-z (PMC7912506; doi:10.1186/s12889-021-10462-z)
Supplement: Supplementary file 4 — Additional file 4. HERStory Qualitative Study Codebook. Pre-determined codebook used for analysis of qualitative data [file 12889_2021_10462_MOESM4_ESM.pdf]

Parent Node	Child Node	Definition	Comments / Instructions
**EFFECT**		Anything about the effect that something has	Only use if child nodes do not apply
	Change	Anything to do with change, changing	Double code with what is changing
	Improve / Benefit	Anything to do with improving, benefiting, having a positive effect on	Double code with what is being spoken about / referred to
	Worsen / Harm	Anything to do with harm, worsening, making worse, having a negative effect on	
	Facilitate	Includes any discussion of facilitators, factors that make something easier, enabling	
	Prevent	Includes any discussion of challenges and barriers, factors that make something more difficult	
**CONTEXT**		Anything about context	
	Social	Anything about social context, cultural context, socio-cultural norms etc	
	Religion	Anything about religion, religious institutions, faith based groups etc	
	Political	Anything about political context	
**ECONOMIC**		Anything relating to economic factors	
	Work	Anything to do with work, employment, working, being employed, jobs, career	
	Income	Anything to do with income, income generation, earning, being paid, receiving money	Double code with SEX for sex work related discussions
	Poverty	Anything to do with poverty, being poor, lack of money or resources, conditions of poverty	

	Grant	Anything to do with social grants	
	Unemployment	Anything to do with not being employed, looking for work, issues related to being unemployed	
	Funding	Anything about funding, financing something, receiving or giving funding	
	Money	Anything to do with money, cash	
**EDUCATION**		Anything about education, learning, school, university, college	Only use if child nodes do not apply
	Attendance	Anything about attending school, including lack of attendance, skipping school, dropping out of school	
	Grades	Anything about grades, performance, results, marks	
	Homework	Anything about homework	
	Exams	Anything about exams, tests	
**EMOTIONS**		Anything to do with how someone feels about something, how they felt in a situation	
	Like	Anything about liking something	Double code with what is being referred to
	Dislike	Anything about disliking something	Double code with what is being referred to
	Happy	Anything about being happy, feeling happy, happiness, joy	
	Sad	Anything about being sad, feeling depressed, depression	Double code with mental health if talking about serious/clinical depression
	Hope	Anything about feeling hopeful, optimistic, dreams, hoping for something. Also included feeling hopeless.	Double code with what the hopes are about (e.g. employment)
	Expectation	Anything to do expectations, includes expectations being met or not being met, includes disappointment	
	Trust	Anything about trust, trusting someone, building trust. Also includes lack of trust, mistrust	

**GLOBAL FUND**		Anything about Global Fund programmes, interventions, and activities, including health services, health education, parenting programmes, career jamborees, homework support, home visits, programmes for perpetrator boys etc	Only use if child nodes do not apply / are not referred to specifically
	Keeping Girls in School	Anything related to Keeping Girls in School programmes	Only use if referred to explicitly – otherwise apply parent node
	RISE Clubs	Anything related to Rise Clubs. Also includes Women of Worth, Rise in and Rise Out	Only use if referred to explicitly – otherwise apply parent node
	Soul Buddyz	Anything related to Soul Buddyz programmes	Only use if referred to explicitly – otherwise apply parent node
	Implementation	Anything relating to the implementation of GF programmes and interventions	
	Integration	Anything relating to the way GF progs and interventions were integrated / not integrated into existing structures and systems	
	Planning	Anything about the planning phase of GF progs and interventions	
	Partnership	Anything about partnership between the GF and other stakeholders, collaboration, working together with people / organisations	
	Participation	Anything about people who participated in the GF progs, including club members	
	Retention	Anything about retention of participants in the GF progs and activities. Including attrition, lack of retention.	
	Sustainability	Anything about the sustainability of programmes, interventions	
**GOVERNMENT**		Any discussion of government, govt departments, govt ministers. Includes discussion of National, Provincial, District level govt. Includes discussion of government policies.	Only use if child nodes do not apply / are not referred to specifically
	Department of Education	Anything about DoE	Only use if referred to explicitly – otherwise apply parent node
	Department of Health	Anything about DoH	Only use if referred to explicitly – otherwise apply parent node

	Service Provision	Anything about services provided by government entities, departments, facilities	Double code with HEALTH for health services
**HEALTH**		Anything about health in general, well-being	Only use if child nodes do not apply. For discussion of health education – double code HEALTH and EDUCATION
	HIV	Anything about HIV, including HIV testing, counselling, treatment, ARVs	
	Mental Health	Anything about mental health, includes discussion around depression, anxiety, suicide	
	Sexual and Reproductive Health	Anything about SRH, includes pregnancy, STIs, contraception, childbirth, abortion, menstruation	
	TB	Anything about TB, including screening, testing, treatment	
**PEOPLE**			
	Friends/Peers	Anything about friends, peers, peer groups	
	Women / Girls	Anything about women, girls, females, femininity, female gender norms/roles etc	
	Men / Boys	Anything about men, boys, males, masculinity, male gender norms/roles	
	Family	Anything about family, including parents, siblings, grandparents, aunts, uncles, cousins, children etc	
	Partner	Anything about partners, boyfriends, girlfriends, husbands, wives. Includes steady partners and casual partners.	
	Educators	Anything about teachers, educators, principals, club facilitators	
	Healthcare workers	Anything about health care providers, doctors, nurses, counsellors	
	Other Organisations	Anything about other organisations / NGOs / NPOs, CBOs, charities, includes FBOs	For FBOs double code with Religion.<br>For CBOs double code with Community
**PLACES**		Anything about physical spaces, spatial aspects	Only use when child node does not apply.

	Community	Anything about the spatial / physical aspect of the neighbourhood or community. Also anything about people in the community, community members, community attitudes	
	Home	Anything about home, the household. Includes living conditions, types of dwelling	
	Clinic	Anything about clinics, health facilities, hospitals, mobile clinics	
	Place of worship	Anything about the church, mosque, temple, or another place of worship	
	School	Anything about the spatial or physical aspects of: school, places of learning, college, university	
**SEX**		Anything about sex, sexual intercourse, including vaginal, anal, oral. Also includes discussion of prevention methods and contraception, including male condoms, female condoms, PrEP, lubricants	Only use when child node does not apply. Double code with income if talking about sex work.
	Sexuality	Anything about sexuality, sexual identity, sexual orientation, being heterosexual, homosexual, bisexual etc	
**BEHAVIOUR**		Anything about behaviour, the way someone behaves or acts	Only use when child node does not apply.
	Decision making	Anything about the process of making decisions, deciding something	Double code with what is being decided on (e.g. SEX)
	Substance Use	Anything about using substances, including alcohol, drinking, smoking, tik etc	
**RELATIONSHIPS**		Anything about relationships between people, includes friendships, marriages, partnerships, working relationships, collaborations, building relationships	
	Love	Anything about love, romance, feeling of love, being in love, romantic feelings, being attracted to someone	
	Power	Anything about power dynamics in relationships, power between people, unequal power, subordination, dominance	Double code with whom (e.g. partner, teacher etc). Double-code with men / women and with Social (CONTEXT) if referring to gendered power
	Gossip	Anything about	

	Stigma / Discrimination	Anything about stigma, being stigmatised, discrimination, being discriminated or experiencing prejudice. Also includes gossip & rumours	
	Support	Anything about being supported, giving support, feeling supported, giving advice, advising someone, giving guidance, help	Double code with who is giving / receiving support, and in what area (e.g. education / financial)
**SAFETY**		Anything about safety and security. Includes feeling safe, secure. As well as feeling unsafe or insecure	
	Risk	Anything about being at risk, feeling at risk, risk-taking, putting yourself at risk	Double code with HIV if talking about HIV risk behaviours. Double-code with SEX if talking about unprotected sex or sexual risk
	Violence	Anything related to any type of violence, harm or abuse: domestic, sexual, psychological, emotional, physical, IPV, gender based violence or rape. Includes actual experiences or discussion of potential risk) in relation to anyone (i.e. participant, partner, family, community members, etc.). Includes being or feeling forced to do something.	
